# Processivity of dimeric kinesin‐1 molecular motors

**DOI:** 10.1002/2211-5463.12486

**Published:** 2018-07-20

**Authors:** Si‐Kao Guo, Xiao‐Xuan Shi, Peng‐Ye Wang, Ping Xie

**Affiliations:** ^1^ Key Laboratory of Soft Matter Physics Beijing National Laboratory for Condensed Matter Physics Institute of Physics Chinese Academy of Science Beijing China; ^2^ School of Physical Sciences University of Chinese Academy of Sciences Beijing China

**Keywords:** coordination, kinesin, mechanochemical coupling, molecular motor, run length

## Abstract

Kinesin‐1 is a homodimeric motor protein that can move along microtubule filaments by hydrolyzing ATP with a high processivity. How the two motor domains are coordinated to achieve such high processivity is not clear. To address this issue, we computationally studied the run length of the dimer with our proposed model. The computational data quantitatively reproduced the puzzling experimental data, including the dramatically asymmetric character of the run length with respect to the direction of external load acting on the coiled‐coil stalk, the enhancement of the run length by addition of phosphate, and the contrary features of the run length for different types of kinesin‐1 with extensions of their neck linkers compared with those without extension of the neck linker. The computational data on other aspects of the movement dynamics such as velocity and durations of one‐head‐bound and two‐head‐bound states in a mechanochemical coupling cycle were also in quantitative agreement with the available experimental data. Moreover, predicted results are provided on dependence of the run length upon external load acting on one head of the dimer, which can be easily tested in the future using single‐molecule optical trapping assays.

**Abbreviations**
[ATP]: ATP concentration
1HB: one‐head‐bound
2HB: two‐head‐bound
ADP: adenosine diphosphate
AMP‐PNP: adenylyl‐imidodiphosphate
ATP: adenosine triphosphate
ATPase: adenosine triphosphatase
ATPγS: adenosine 5′‐*O*‐(3‐thiotriphosphate)
CL: cysteine‐light
DmK‐6AA: *Drosophila* kinesin‐1 with six additional amino acid residues inserted into the C‐terminal portion of the linker region of each head
DmK‐WT: wild‐type *Drosophila* kinesin‐1
HsK‐CL: cysteine‐light human kinesin‐1
HsK‐CL‐6AA: cysteine‐light human kinesin‐1 with six additional amino acid residues inserted into the C‐terminal portion of the linker region of each head
MD: molecular dynamics
MT: microtubule
NL: neck linker
P_i_: phosphate

Conventional kinesin (kinesin‐1) is a homodimeric motor protein that can move processively along microtubule (MT) filaments to transport cargo within cells by hydrolyzing ATP 1. It is a model system for studying biological molecular motors that can move processively on linear tracks. To understand the molecular mechanism of kinesin‐1's processive movement, besides structural studies 2, 3, 4, various experimental methods have been employed to study its movement dynamics 5, 6, 7, 8, 9, 10. In particular, using single‐molecule optical trapping, many aspects of its dynamics, such as the velocity, stall force, and mechanochemical coupling ratio, have been studied elaborately 11, 12, 13, 14, 15, 16, 17, 18, 19, 20, 21. The processivity, which is characterized by the run length (i.e. the distance traveled by an individual motor on its liner track before dissociating), is another important factor that characterizes the dynamics of a molecular motor.

Recently, using high‐resolution single‐molecule optical trapping techniques, Milic *et al*. 22 measured systematically the run length of *Drosophila* kinesin‐1 dimer under both hindering (backward) and assisting (forward) loads. Interestingly, they found that the addition of phosphate in the solution enhanced the run length. Even more interestingly, the experimental data showed a dramatic asymmetry of the run length with respect to the direction of the external load acting on the coiled‐coil stalk 22, 23. Under a moderate forward load, the run length was an order of magnitude shorter than under the corresponding backward load 22, 23. Furthermore, the sensitivity of the run length to load, as characterized by a distance parameter, was dramatically lower in the forward‐load regime than in the backward‐load regime 22, 23. These results are very puzzling. According to Kramers theory, the dependence of a motor's unbinding rate, ε, on the external force, *F*, is calculated by ε = ε_0_exp(|*F*|/|*F*_d_), where ε_0_ is the unbinding rate under no force and *F*_d_ is the unbinding force. On the other hand, the experimental data showed that the movement velocity, *v*, decreases with the increase in the magnitude of the backward force but is nearly independent of the magnitude of the forward force 23. Thus, it would be expected that a backward force causes a larger decrease in the run length (*L *= *v*/ε) than a forward force of the same magnitude, which is contrary to the experimental data 22, 23. Additionally, Andreasson *et al*. 23 found that by extending the neck linkers (NLs) of *Drosophila* kinesin‐1 dimer, the run length under no load was reduced greatly compared with the wild‐type case, whereas the NL extension only causes a small reduction in the velocity. On the contrary, for a cysteine‐light (CL) human kinesin‐1 construct, where a residue cysteine in the NL domain was mutated, the extension of the NLs has an insensitive effect on the run length or even increases the run length under no load, while the extension causes a large reduction in the velocity 24, 25. However, even qualitative explanations of the above puzzling experimental data have not been presented up to now. How does *Drosophila* kinesin‐1 dimer show the dramatically asymmetric character of the run length with respect to the direction of the external load? By extension of their NLs, why do different types of kinesin‐1 dimer behave rather differently or contrarily in their run length and velocity compared with those with no extension of the NLs? As is known, the dissociation of the kinesin dimer from MT, which determines the run length, involves the coordination of the two heads of the dimer during the processive stepping, and the coordination involves the mechanochemical coupling of the dimer. Thus, quantitative explanations of these experimental data on the run length and, in particular, the dramatic asymmetry of the run length with respect to the direction of the external load and the rather different characters of the run length for different types of dimers with extensions of the NLs have important implications for the mechanochemical coupling mechanism of the dimer.

Theoretically and computationally, the mechanism and dynamics of the processive movement of kinesin‐1 dimers have also been studied extensively 26, 27, 28, 29, 30, 31, 32, 33, 34, 35, 36. More recently, a model for the processive movement of the dimeric kinesin was presented 37. With the model, diverse experimental data on the movement dynamics of the dimeric kinesin‐1 were explained quantitatively, such as the effects of varying solution viscosity, varying the external loads acting on the bead attached to the coiled‐coil stalk and on the bead attached to one head of the dimer, nullifying the NL docking, and extending the NLs on the velocity, mechanochemical coupling ratio, stall force, and randomness parameter 37. Moreover, the experimentally observed asymmetric or limping stepping dynamics of the homodimer and different features on the velocity *versus* the external load between different types of the kinesin‐1 dimer were also explained quantitatively 38.

In this work, we improved the model presented previously 37, 38. With the improved model, we studied computationally the processivity of the kinesin‐1 dimer, to understand the detailed mechanochemical coupling mechanism. The computational data reproduce well diverse experimental data on the run length and the corresponding velocity, providing a consistent and quantitative explanation of the above‐mentioned puzzling experimental data, as well as other experimental data such as the durations of one‐head‐bound (1HB) and two‐head‐bound (2HB) states in one step of the processive movement of the dimer, and the effect of additional phosphate on run length. Furthermore, to further test the model, some predicted results are provided.

## Materials and methods

### The model

The model of processive movement of dimeric kinesin is described as follows, which is an improvement on that proposed before 37. It is built up based mainly on three pieces of experimental and computational evidence and/or arguments.

**Figure 1:**
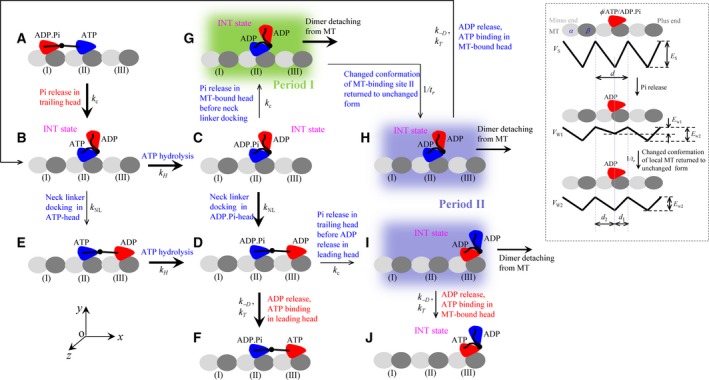
Schematic illustrations of the model of a typical forward stepping of the dimer at saturating ATP. Inset shows interaction potentials between the single kinesin head and MT along a MT protofilament during an ATPase cycle, with the top panel showing the strong interaction potential, *V*_S_, in nucleotide‐free (φ), ATP, or ADP.P_i_ states; the middle panel showing the weak interaction potential, *V*_W__1_, in ADP state immediately after P_i_ release; and the bottom panel showing the weak interaction potential, *V*_W__2_, in ADP state in a period of time *t*_r_ after P_i_ release. (A) The trailing head in ADP.P_i_ state binds strongly to the rear MT‐binding site (I) while the leading head in ATP state binds strongly to the forward MT‐binding site (II). The trailing head with its NL pointing forward has a larger P_i_ release rate than the leading head with its NL not pointing forward. (B) Upon P_i_ release in the trailing head, due to the very weak affinity (*E*_w1_) between the ADP–head and the local MT‐binding site (I), driven by the thermal noise, the trailing ADP–head diffuses rapidly to the intermediate position relative to the other MT‐bound head, where the two heads have strong affinity. (C) ATP is hydrolyzed to ADP.P_i_ in the MT‐bound head. (D) In the intermediate state with the MT‐bound head in ADP.P_i_ state, the NL docking takes place, weakening the interaction between the two heads. Then, the thermal noise drives the tethered head to diffuse rapidly to the forward MT‐binding site (III). (E) In the intermediate state of (B), before ATP is hydrolyzed to ADP.P_i_ in the MT‐bound head, NL docking can also take place with a very low probability due to the very slow rate of NL docking in ATP state, weakening the interaction between the two heads. The tethered head then diffuses rapidly to the forward MT‐binding site (III). (F) Stimulated by MT ADP is released rapidly, followed by ATP binding. (G) From (C), P_i_ release can also occur occasionally in the MT‐bound head before its NL docking. Before the affinity of the MT‐bound ADP–head for the local MT‐binding site (II) changes from *E*_w1_ to *E*_w2_, the dimer can easily detach from MT by overcoming the very weak affinity *E*_w1_. (H) From (G) the affinity of the MT‐bound ADP–head for the local MT‐binding site (II) changes from *E*_w1_ to *E*_w2_ in time *t*_r_. The dimer also has a large probability to detach from MT before ADP release from the MT‐bound head by overcoming weak affinity *E*_w2_. If the dimer has not detached until ADP release, which is followed by ATP binding, the system returns to (B). (I) From (D), P_i_ release can also occur occasionally in the trailing head before ADP release in the leading head. The dimer has a large probability to detach from MT before ADP release from the MT‐bound head by overcoming weak affinity *E*_w2_. (J) From (I), the dimer has not detached until ADP release, which is followed by ATP binding. The thickness of the arrow represents the magnitude of the transition probability under no load. The states (shaded in green and blue) where the dimer can detach from MT are indicated.
(b) The available experimental data showed that the NL can be docked into its motor domain in a nucleotide‐dependent manner 46. Based on the experimental data 46, 47, we make the following argument on this nucleotide‐dependent NL docking. When an MT‐bound kinesin head is in the nucleotide‐free and ADP states, the NL is unable to dock. When the head is in the ADP.P_i_ state, there is a small free energy (denoted by *E*_NL_) to facilitate efficiently its NL docking. The docking involves N‐terminal strand β0 of the motor domain forming a cover–neck bundle with strand β9 of the NL 48, which occurs when the two strands are in proximity. (See Section S2 for a mathematical description of the potential of the effect of NL docking into the MT‐bound head on the movement of the other tethered ADP–head.) In the ATP state, the NL has a lower efficiency or rate of docking than in the ADP.P_i_ state, as fluorescence polarization microscopy data showed 47. In the Discussion, we will further discuss the nucleotide‐dependent NL docking.
(c) We argue that there exists an interaction between the two heads. When the NLs of both heads are undocked, the two heads have a high binding energy (denoted by *E*_I1_), and the NL docking in one head weakens the interaction between the two heads, with the binding energy denoted by *E*_I2_ < *E*_I1_. (See Section S3 for a mathematical description of the potential of the interaction between the two kinesin heads.) Note that with this argument together with argument (b), the biochemical data of Hackney 49, showing that upon the dimer with both heads bound by ADP mixing with MT only one‐half of the ADP molecules are released and addition of ATP molecules leads to release of other half of the ADP molecules, can be explained well.

(a) The experimental data showed that the kinesin head in the nucleotide‐free, ATP, or ADP.P_i_ state has a strong interaction with MT, whereas in the ADP state, it has a weak interaction 39, 40, 41, 42, 43, 44. The structural data showed that the strong interaction of MT tubulin heterodimer with the kinesin head in the strong MT‐binding state induces large conformational changes in the tubulin heterodimer 45. The recent all‐atom molecular dynamics (MD) simulations showed that the MT tubulin heterodimer with such conformational changes has a much weaker binding energy to the ADP–head than unperturbed MT tubulin heterodimers (X‐X. Shi, Y‐B. Fu, S‐K. Guo, P‐Y. Wang, H. Chen, P. Xie, under review). Thus, it is argued that immediately after the P_i_ release, the binding energy of the ADP–head with the local MT‐binding site (denoted by *E*_w1_), where the head in the strong MT‐binding state has just bound, is temporarily weaker than that with other unperturbed MT‐binding sites (denoted by *E*_w2_ > *E*_w1_) 35, 36, 37, 38. In a time *t*_r_, the local tubulin relaxes to its normally unperturbed conformation, with the interaction energy of the ADP–head with the local tubulin becoming the same as that with other tubulins. As a result, the interaction potential between the head and MT along a MT protofilament in an ATPase cycle is approximately shown in the inset of Fig. 1. (See Section S1 in Supporting information for a mathematical description of the interaction potential.)

Based on the three pieces of experimental evidence and/or arguments, a typical forward stepping of the dimer at saturating ATP is schematically shown in Fig. 1. Before stepping, the trailing head in the ADP.P_i_ state binds strongly to the rear MT‐binding site (I), while the leading head in the ATP state binds strongly to the forward MT‐binding site (II) (the available single‐molecule data indicating that ATP can bind to the leading head when the trailing head is bound strongly to MT 25) (Fig. 1A). The trailing head with its NL pointing forward has a larger P_i_ release rate than the leading head with its NL not pointing forward (the biochemical evidence for this characteristic is discussed in the section ‘The choice of parameter values’). Upon P_i_ release in the trailing head (with a rate constant *k*_c_), due to the very weak affinity (*E*_w1_) between the ADP–head and the local MT‐binding site (I), driven by the thermal noise, the trailing ADP–head detaches from site (I) and then diffuses rapidly to the intermediate position relative to the other MT‐bound head, where the two heads bind together strongly (Fig. 1B) (the state of the dimer with one head bound to MT and the other ADP–head bound to the MT‐bound head is called the intermediate state or 1HB state). ATP is hydrolyzed to ADP.P_i_ in the MT‐bound head (with a rate constant *k*_H_) (Fig. 1C). In the intermediate state (Fig. 1C), with the MT‐bound head in ADP.P_i_ state and strand β9 of the NL being close to strand β0 of the motor domain, NL docking takes place efficiently by forming the cover–neck bundle (with a high rate constant *k*_NL_). The docking weakens the interaction between the two heads. Then, the thermal noise drives the tethered ADP–head to diffuse rapidly to the nearest forward MT‐binding site (III) (Fig. 1D). Note here that the NL docking in the MT‐bound head provides an energy barrier *E*_NL_ to prevent the tethered ADP–head from moving backward to the rear MT‐binding site (I). In the intermediate state (Fig. 1B), before ATP is hydrolyzed to ADP.P_i_ in the MT‐bound head, NL docking can also take place with a very low probability due to the slow rate of NL docking in the ATP state, weakening the interaction between the two heads. The thermal noise then drives the tethered ADP–head to diffuse rapidly to the nearest forward MT‐binding site (III) (Fig. 1E), which is followed by ATP hydrolysis to ADP.P_i_ in the trailing head (with the rate constant *k*_H_) (Fig. 1D). After the ADP–head binds to the forward MT‐binding site (III) (Fig. 1D), stimulated by MT, ADP is released rapidly (with a rate constant *k*_–D_) and ATP of saturating concentration then binds immediately to the nucleotide‐free head (with a rate constant *k*_T_) (Fig. 1F).

It can be noted that in the intermediate state of Fig. 1C, P_i_ release can also occur occasionally in the MT‐bound head before its NL docking takes place (Fig. 1G). In Fig. 1G, before the affinity of the MT‐bound ADP–head for the local MT‐binding site (II) changes from *E*_w1_ to *E*_w2_, the dimer can easily detach from MT by overcoming the very weak affinity *E*_w1_. If the dimer has not detached from MT until the affinity of the MT‐bound ADP–head for the local MT‐binding site (II) changed from *E*_w1_ to *E*_w2_ in time *t*_r_ (Fig. 1H), the dimer also has a large probability to detach from MT before ADP release from the MT‐bound head by overcoming weak affinity *E*_w2_ (Fig. 1H). If the dimer has not detached until ADP release, the system returns to that of Fig. 1B. Note also that in Fig. 1D, P_i_ release can also occur occasionally in the trailing head before ADP release in the leading head (Fig. 1I). In the intermediate state of Fig. 1I, the dimer also has a large probability to detach from MT before ADP release from the MT‐bound head by overcoming weak affinity *E*_w2_. If the dimer has not detached until ADP release, the system transits to that of Fig. 1J, which is the same as that of Fig. 1B except that the dimer has moved a step forward.

### Equations to describe the movement of tethered ADP–head relative to nucleotide‐free head, ATP–head, or ADP.P_i_–head bound fixedly to MT

In this work, for simplicity of analysis, we do not consider the dissociation of the nucleotide‐free head, ATP–head, or ADP.P_i_–head from MT, because in nucleotide‐free, ATP, and ADP.P_i_ states, the kinesin head binds to MT strongly. In this section, we present equations for the movement of the ADP–head relative to the nucleotide‐free head, ATP–head, or ADP.P_i_–head bound fixedly to MT. We define the coordinate *oxyz* as shown in Fig. 1, where the origin of the coordinate (0,0,0) is at the center‐of‐mass position of the MT‐bound head. We consider the translation motion of the ADP–head in three dimensions (denoted by coordinates *x*,* y*, and *z*) and rotation in three directions. The rotation is described by nutation motion (characterized by angle α), rotation motion (characterized by angle θ), and precession motion (characterized by angle *ϕ*). When the kinesin head is in the MT‐binding site, α, θ, and *ϕ* correspond to the angles of rotation in the *xoz*,* xoy*, and *yoz* planes, respectively.

As done in the single‐molecule optical trapping experiments 22, we consider a micrometer‐sized bead with diameter of 2*R*_bead_ attached to the coiled‐coil stalk of the dimer and an external force acting on the bead. With one kinesin head binding fixedly to MT at position (0,0,0), the translation and rotation of the other ADP–head relative to the MT‐bound head in viscous solution can be described by Langevin equations 37, 38

(1) $$\Gamma_{x}\frac{\partial x}{\partial t}=-\frac{\partial V_{W}\left(x,y,z,\alpha ,\theta ,\phi \right)}{\partial x}-\frac{\partial V_{I}\left(x,y,z,\alpha ,\theta ,\phi \right)}{\partial x}-\frac{\partial V_{\mathrm{NL}}\left(x\right)}{\partial x}-F_{\mathrm{NL}}\left(\frac{r}{2}\right)\frac{x}{r}+\xi_{x}\left(t\right),\text{when}|x-x_{\mathrm{bead}}\left|<\right|x_{\mathrm{bead}}|,$$

(2) $$\begin{array}{cc} \Gamma_{x}\frac{\partial x}{\partial t}= & -\frac{\partial V_{W}\left(x,y,z,\alpha ,\theta ,\phi \right)}{\partial x}-\frac{\partial V_{I}\left(x,y,z,\alpha ,\theta ,\phi \right)}{\partial x}-\frac{\partial V_{\mathrm{NL}}\left(x\right)}{\partial x}\\ & -F_{\mathrm{NL}}\left(\frac{r}{2}\right)\frac{x}{r}-C\left[\left(x-x_{\mathrm{bead}}\right)-\text{sgn}\left(x-x_{\mathrm{bead}}\right)\frac{d}{2}\right]\\ & H\left(|x-x_{\mathrm{bead}}|-\frac{d}{2}\right)+\xi_{x}\left(t\right),\;\text{when}|x-x_{\mathrm{bead}}\left|\geq \right|x_{\mathrm{bead}}|, \end{array}$$

(3) $$\begin{array}{cc} \Gamma_{x}^{\left(\mathrm{bead}\right)}\frac{\partial x_{\mathrm{bead}}}{\partial t}= & C\left[\left(0-x_{\mathrm{bead}}\right)-\text{sgn}\left(0-x_{\mathrm{bead}}\right)\frac{d}{2}\right]\\ & H\left(|x_{\mathrm{bead}}|-\frac{d}{2}\right)+F_{x}+\xi_{x}^{\left(\mathrm{bead}\right)}\left(t\right),\\ & \text{when}|x-x_{\mathrm{bead}}\left|<\right|x_{\mathrm{bead}}|, \end{array}$$

(4) $$\begin{array}{cc} \Gamma_{x}^{\left(\mathrm{bead}\right)}\frac{\partial x_{\mathrm{bead}}}{\partial t}= & \;C\left[\left(x-x_{\mathrm{bead}}\right)-\text{sgn}\left(x-x_{\mathrm{bead}}\right)\frac{d}{2}\right]\\ & H\left(|x-x_{\mathrm{bead}}|-\frac{d}{2}\right)+F_{x}+\xi_{x}^{\left(\mathrm{bead}\right)}\left(t\right),\\ & \text{when}\;|x-x_{\mathrm{bead}}\left|\geq \right|x_{\mathrm{bead}}|, \end{array}$$

(5) $$\Gamma_{y}\frac{\partial y}{\partial t}=-\frac{\partial V_{W}\left(x,y,z,\alpha ,\theta ,\phi \right)}{\partial y}-\frac{\partial V_{I}\left(x,y,z,\alpha ,\theta ,\phi \right)}{\partial y}-F_{\mathrm{NL}}\left(\frac{r}{2}\right)\frac{y}{r}+C\left(y_{\mathrm{bead}}-y\right)+\xi_{y}\left(t\right),\text{when}\;y<1\text{nm},$$

(6) $$\Gamma_{y}\frac{\partial y}{\partial t}=-\frac{\partial V_{W}\left(x,y,z,\alpha ,\theta ,\phi \right)}{\partial y}-\frac{\partial V_{I}\left(x,y,z,\alpha ,\theta ,\phi \right)}{\partial y}-F_{\mathrm{NL}}\left(\frac{r}{2}\right)\frac{y}{r}+\xi_{y}\left(t\right),\text{when}\;y\geq 1\text{nm},$$

(7) $$\Gamma_{y}^{\left(\mathrm{bead}\right)}\frac{\partial y_{\mathrm{bead}}}{\partial t}=C\left[\left(y-y_{\mathrm{bead}}\right)+\left(0-y_{\mathrm{bead}}\right)\right]+F_{y}+\xi_{y}^{\left(\mathrm{bead}\right)}\left(t\right),\text{when}\;y<1\text{nm},$$

(8) $$\Gamma_{y}^{\left(\mathrm{bead}\right)}\frac{\partial y_{\mathrm{bead}}}{\partial t}=C\left[(0-y_{\mathrm{bead}})\right]+F_{y}+\xi_{y}^{\left(\mathrm{bead}\right)}\left(t\right),\text{when}\;y\geq 1\text{nm},$$

(9) $$\Gamma_{z}\frac{\partial z}{\partial t}=-\frac{\partial V_{W}\left(x,y,z,\alpha ,\theta ,\phi \right)}{\partial z}-\frac{\partial V_{I}\left(x,y,z,\alpha ,\theta ,\phi \right)}{\partial z}-F_{\mathrm{NL}}\left(\frac{r}{2}\right)\frac{z}{r}+\xi_{z}\left(t\right),$$

(10) $$\Gamma_{\alpha }\frac{\partial \alpha }{\partial t}=-\frac{\partial V_{W}\left(x,y,z,\alpha ,\theta ,\phi \right)}{\partial \alpha }-\frac{\partial V_{I}\left(x,y,z,\alpha ,\theta ,\phi \right)}{\partial \alpha }+\xi_{\alpha }\left(t\right),$$

(11) $$\Gamma_{\theta }\frac{\partial \theta }{\partial t}=-\frac{\partial V_{W}\left(x,y,z,\alpha ,\theta ,\phi \right)}{\partial \theta }-\frac{\partial V_{I}\left(x,y,z,\alpha ,\theta ,\phi \right)}{\partial \theta }+\xi_{\theta }\left(t\right),$$

(12) $$\Gamma_{\phi }\frac{\partial \phi }{\partial t}=-\frac{\partial V_{W}\left(x,y,z,\alpha ,\theta ,\phi \right)}{\partial \phi }-\frac{\partial V_{I}\left(x,y,z,\alpha ,\theta ,\phi \right)}{\partial \phi }+\xi_{\phi }\left(t\right),$$

where $r=\sqrt{x^{2}+y^{2}+z^{2}}$, and *x*_bead_ and *y*_bead_ denote, respectively, the *x*‐coordinate and *y*‐coordinate of the center of mass of the bead (for simplicity but without loss of generality, the motion of the bead in the *z*‐coordinate is not considered). *V*_W_ (*x*,* y*,* z*, α, θ, *ϕ*) is the potential of the ADP–head interacting with the MT‐binding site during the period after P_i_ release and before ADP release with the other nucleotide‐free head, ATP–head, or ADP.P_i_–head bound fixedly to MT (see Section S1), *V*_NL_(*x*) is the potential characterizing the effect of the NL docking to the MT‐bound head on the motion of the tethered ADP–head (see Section S2), *V*_I_ (x, y, z, α, θ, *ϕ*) is the potential of interaction between the two kinesin heads (see Section S3), and *F*_NL_ is the force acting on the ADP–head that results from the stretching of the NLs (see Section S4).

In Equations ((1), (2), (3), (4), (5), (6), (7), (8), (9), (10), (11), (12)), we take the followings into consideration. Due to the steric restriction of MT and considering the size of the kinesin head with radius *r*_head_ = 2.5 nm, it is required that *y ≥* *y*_0_ = 0 and *r ≥ *2*r*_head_ = 5 nm. Due to the steric restriction of MT and considering the size of the bead with radius *R*_bead_, it is required that *y*_bead_ ≥ *R*_bead_. For the longitudinal or *x* component of the external force, *F*_*x*_, the vertical or *y* component is calculated by *F*_*y*_ = |*F*_*x*_|tanΘ_0_ and sinΘ_0_ = *R*_bead_/(*R*_bead_ + *l*_kinesin_), where *l*_kinesin_ = 54 nm is the length of the coiled‐coil stalk of the kinesin dimer 38 and 2*R*_bead_ = 0.44 μm as used in the experiments 22. *F*_*x*_ is defined as negative when it points backward (i.e. the –*x* direction) and positive when it points forward (i.e. the +*x* direction), while *F*_*y*_ is defined as positive when it points upward (i.e. the +*y* direction). It is considered that the *x* component of the interaction force between the bead and kinesin dimer acts only on the NL of the head that has a larger distance to the bead along the *x* direction. Thus, when the distance between a kinesin head and bead along the *x* direction is smaller than *d*/2, no *x* component of the internally elastic force exists between them, with *d *=* *8.2 nm being the distance between two successive binding sites along a MT protofilament. Function sgn(*x*) is the sign function, and function *H*(*x*) is defined as follows: *H*(*x*) = 1 if *x *>* *0 and *H*(*x*) = 0 if *x ≤ *0. It is noted that when the two heads are bound simultaneously to MT, each head experiences a vertical force because the two heads have the same distance to the bead along the *y* direction. When only one head is bound to MT and the other head is detached from MT, only the MT‐bound head experiences a vertical force, because the distance of the bead to the MT‐bound kinesin head along the vertical *y* direction is larger than that to the detached kinesin head. For approximation, we consider here that when the ADP–head is in the range of 0 ≤ *y *<* *1 nm (noting that when bound to MT, the head is in the position of *y *=* *0), the ADP–head experiences the vertical force, and when in other ranges (*y *≥* *1 nm), the ADP–head experiences no vertical force. The drag coefficients on the kinesin head are calculated by Γ_*x*_ = Γ_*y*_ = Γ_*z*_ = 6πη_0_ *r*_head_ and Γ_α_ = Γ_θ_ = Γ_φ_ = 8πη_0_ $r_{\mathrm{head}}^{3}$, where η_0_ is the solution viscosity in the vicinity of MT. Since the viscosity in the vicinity of MT is larger than that far away from MT, we take η_0_ = 0.02 g·cm^−1^·s^−1^, which is about twofold larger than that in water. The term ξ_*i*_(*t*) (*i *= *x*,* y*,* z*, α, θ, *ϕ*) is the fluctuating Langevin force on the kinesin head, with $\left\langle \xi_{i}\left(t\right)\right\rangle =0,\left\langle \xi_{i}\left(t\right)\xi_{j}\left(t^{\prime }\right)\right\rangle =0$ (*i* ≠ *j*) and $\left\langle \xi_{i}\left(t\right)\xi_{i}\left(t^{\prime }\right)\right\rangle =2k_{B}T\Gamma_{i}\delta \left(t-t^{\prime }\right)$, where *k*_B_ is the Boltzmann constant and *T* is absolute temperature. The drag coefficients on the bead are calculated by $\Gamma_{x}^{\left(\mathrm{bead}\right)}=\Gamma_{y}^{\left(\mathrm{bead}\right)}$ = 6πη_0_R_bead_. The terms $\xi_{x}^{\left(\mathrm{bead}\right)}\left(t\right)$ and $\xi_{y}^{\left(\mathrm{bead}\right)}\left(t\right)$ are the fluctuating Langevin forces on the bead along the *x* and *y* directions, respectively, with $\langle \xi_{j}^{\left(\mathrm{bead}\right)}\left(t\right)\rangle =0$ (*j *= *x*,* y*), $\langle \xi_{x}^{\left(\mathrm{bead}\right)}\left(t\right)\xi_{y}^{\left(\mathrm{bead}\right)}\left(t\right)\rangle =0$ and $\left\langle \xi_{j}^{\left(\mathrm{bead}\right)}\left(t\right)\xi_{j}^{\left(\mathrm{bead}\right)}\left(t^{\prime }\right)\right\rangle =2k_{B}T\Gamma_{j}^{\left(\mathrm{bead}\right)}\delta \left(t-t^{\prime }\right)$. The connection between the bead and C‐terminal ends of the NLs is characterized by an elastic linear spring with a spring constant *C*. In the calculation, we take *C *=* *0.1 pN·nm^−1^ (we have checked that varying the value of *C* has little effect on the calculated results). The initial conditions for Equations ((1), (2), (3), (4), (5), (6), (7), (8), (9), (10), (11), (12)) are: (*x*_0_, *y*_0_, *z*_0_, α_0_, θ_0_, *ϕ*_0_) = (*−d*, 0, 0, 0, 0, 0), *x*_bead0_ = *−d*/2 + *F*_*x*_/*C*, and *y*_bead0_ = *F*_*y*_/(2*C*).

### Equations to describe the movement of kinesin when two heads are in the ADP state

When both ADP–heads are bound simultaneously to MT, the movement of one head relative to the other can still be described by Equations ((1), (2), (3), (4), (5), (6), (7), (8), (9), (10), (11), (12)). If one head is detached from MT with an affinity of *E*_w1_ (see Fig. 1), it would most probably bind immediately to the other MT‐bound ADP–head due to the high binding energy *E*_I1_ between them because the NL of the MT‐bound ADP–head is undocked. If one head is detached from MT with an affinity of *E*_w2_ (see Fig. 1), it would either rebind immediately to MT or bind immediately to the other MT‐bound ADP–head. When the two ADP–heads are bound together strongly, the movement of the MT‐bound ADP–head relative to MT can be described by the following equations:

(13) $$\begin{array}{cc} \Gamma_{x}\frac{\partial x}{\partial t} & =-\frac{\partial V_{W}\left(x,y,z,\alpha ,\theta ,\phi \right)}{\partial x}\\ & +F_{\mathrm{NL}}\left[\sqrt{{\left(x_{\mathrm{bead}}-x\right)}^{2}+{\left(y_{\mathrm{bead}}-y\right)}^{2}}-\left(R_{\mathrm{bead}}+l_{\mathrm{kinesin}}\right)\right]\\ & \times H\left[\sqrt{{\left(x_{\mathrm{bead}}-x\right)}^{2}+{\left(y_{\mathrm{bead}}-y\right)}^{2}}-\left(R_{\mathrm{bead}}+l_{\mathrm{kinesin}}\right)\right]\\ & \frac{x_{\mathrm{bead}}-x}{\sqrt{{\left(x_{\mathrm{bead}}-x\right)}^{2}+{\left(y_{\mathrm{bead}}-y\right)}^{2}}}+\xi_{x}\left(t\right) \end{array},$$

(14) $$\begin{array}{cc} & \Gamma_{x}^{\left(\mathrm{bead}\right)}\frac{\partial x_{\mathrm{bead}}}{\partial t}=\\ & -F_{\mathrm{NL}}\left[\sqrt{{\left(x_{\mathrm{bead}}-x\right)}^{2}+{\left(y_{\mathrm{bead}}-y\right)}^{2}}-\left(R_{\mathrm{bead}}+l_{\mathrm{kinesin}}\right)\right]\\ & \times H\left[\sqrt{{\left(x_{\mathrm{bead}}-x\right)}^{2}+{\left(y_{\mathrm{bead}}-y\right)}^{2}}-\left(R_{\mathrm{bead}}+l_{\mathrm{kinesin}}\right)\right]\\ & \frac{x_{\mathrm{bead}}-x}{\sqrt{{\left(x_{\mathrm{bead}}-x\right)}^{2}+{\left(y_{\mathrm{bead}}-y\right)}^{2}}}+F_{x}+\xi_{x}^{\left(\mathrm{bead}\right)}\left(t\right) \end{array},$$

(15) $$\begin{array}{cc} \Gamma_{y}\frac{\partial y}{\partial t} & =-\frac{\partial V_{W}\left(x,y,z,\alpha ,\theta ,\phi \right)}{\partial y}\\ & +F_{\mathrm{NL}}\left[\sqrt{{\left(x_{\mathrm{bead}}-x\right)}^{2}+{\left(y_{\mathrm{bead}}-y\right)}^{2}}-\left(R_{\mathrm{bead}}+l_{\mathrm{kinesin}}\right)\right]\\ & \times H\left[\sqrt{{\left(x_{\mathrm{bead}}-x\right)}^{2}+{\left(y_{\mathrm{bead}}-y\right)}^{2}}-\left(R_{\mathrm{bead}}+l_{\mathrm{kinesin}}\right)\right]\\ & \frac{y_{\mathrm{bead}}-y}{\sqrt{{\left(x_{\mathrm{bead}}-x\right)}^{2}+{\left(y_{\mathrm{bead}}-y\right)}^{2}}}+\xi_{y}\left(t\right), \end{array}$$

(16) $$\begin{array}{cc} & \Gamma_{y}^{\left(\mathrm{bead}\right)}\frac{\partial y_{\mathrm{bead}}}{\partial t}=\\ & -F_{\mathrm{NL}}\left[\sqrt{{\left(x_{\mathrm{bead}}-x\right)}^{2}+{\left(y_{\mathrm{bead}}-y\right)}^{2}}-\left(R_{\mathrm{bead}}+l_{\mathrm{kinesin}}\right)\right]\\ & \times H\left[\sqrt{{\left(x_{\mathrm{bead}}-x\right)}^{2}+{\left(y_{\mathrm{bead}}-y\right)}^{2}}-\left(R_{\mathrm{bead}}+l_{\mathrm{kinesin}}\right)\right]\\ & \frac{y_{\mathrm{bead}}-y}{\sqrt{{\left(x_{\mathrm{bead}}-x\right)}^{2}+{\left(y_{\mathrm{bead}}-y\right)}^{2}}}+F_{y}+\xi_{y}^{\left(\mathrm{bead}\right)}\left(t\right) \end{array},$$

(17) $$\Gamma_{z}\frac{\partial z}{\partial t}=-\frac{\partial V_{W}\left(x,y,z,\alpha ,\theta ,\phi \right)}{\partial z}+\xi_{z}\left(t\right),$$

(18) $$\Gamma_{\alpha }\frac{\partial \alpha }{\partial t}=-\frac{\partial V_{W}\left(x,y,z,\alpha ,\theta ,\phi \right)}{\partial \alpha }+\xi_{\alpha }\left(t\right),$$

(19) $$\Gamma_{\theta }\frac{\partial \theta }{\partial t}=-\frac{\partial V_{W}\left(x,y,z,\alpha ,\theta ,\phi \right)}{\partial \theta }+\xi_{\theta }\left(t\right),$$

(20) $$\Gamma_{\phi }\frac{\partial \phi }{\partial t}=-\frac{\partial V_{W}\left(x,y,z,\alpha ,\theta ,\phi \right)}{\partial \phi }+\xi_{\phi }\left(t\right).$$

where *F*_NL_ is the force acting on the MT‐bound ADP–head that results from the stretching of the NLs (see Section S4). Here, for simplicity of treatment, the bead and C‐terminal end of the NL of MT‐bound ADP–head are implicitly considered to be connected rigidly. The initial conditions for Equations ((13), (14), (15), (16), (17), (18), (19), (20)) are as follows: (*x*_0_, *y*_0_, *z*_0_, α_0_; θ_0_, *ϕ*_0_) = (0, 0, 0, 0, 0, 0), *x*_bead0_ = $\left(R_{\mathrm{bead}}+l_{\mathrm{kinesin}}+l_{\mathrm{NL}}\right)F_{x}/\sqrt{F_{x}^{2}+F_{y}^{2}}$ (*x*_bead0_ = 0 when *F*_*x*_ = 0), and *y*_bead0_ = $\left(R_{\mathrm{bead}}+l_{\mathrm{kinesin}}+l_{\mathrm{NL}}\right)F_{y}/\sqrt{F_{x}^{2}+F_{y}^{2}}$ (*y*_bead0_ = *R*_bead_ + *l*_kinesin_ when *F*_*x*_ = *F*_*y*_ = 0), where *l*_NL_ is the length of the NL under a pulling force of magnitude $\sqrt{F_{x}^{2}+F_{y}^{2}}$ .

### Monte Carlo simulations of processive movement and dissociation of dimeric kinesin

Using Equations ((1), (2), (3), (4), (5), (6), (7), (8), (9), (10), (11), (12), (13), (14), (15), (16), (17), (18), (19), (20)), we can simulate the mechanical step of the movement of a kinesin head following P_i_ release relative to the other MT‐bound kinesin head and the dissociation of the dimer from MT using a stochastic Runge–Kutta algorithm, as done before 35, 37, 38, 50. Then, we can simulate processive movement of the dimer by also considering continuous ATPase activities, which can be simulated using a Monte Carlo algorithm, as used before 37, 38, 51. In the Monte Carlo simulations, during each time step Δ*t* (Δ*t* = 10^−4^ s in our simulation), a random number *ran* is generated with uniform probability between 0 and 1. The state transition with rate constant *k*_*i*_ (where *i *represents T, H, c, NL, –D) takes place if *ran ≤ P*_*i*_, and the transition does not take place if *ran* > *P*_*i*_. Here, *P*_*i*_ = *k*_*i*_Δ*t* is the probability of state transition in each time step Δ*t*,* k*_T_ = $k_{b}^{\left(T\right)}$ [ATP] represents ATP binding rate to the nucleotide‐free head, with $k_{b}^{\left(T\right)}$ being the second‐order rate constant for ATP binding and [ATP] being the ATP concentration, *k*_H_ represents the rate constant of ATP hydrolysis, *k*_c_ represents the rate constant of P_i_ release, *k*_NL_ represents the rate constant of NL docking into the motor domain of MT‐bound head in the ATP or ADP.P_i_ state when the detached ADP–head is in the intermediate position, and *k*_–D_ represents the rate constant of ADP releasing from ADP–head. In our simulations, the rate constant *k*_*i*_ (where *i* represents T, H, c, NL, –D) is independent of the strain on the NL (see next section).

As mentioned above, we take the following into consideration to study the dissociation of the dimer from MT. When one head of the dimer in the nucleotide‐free, ATP, or ADP.P_i_ state binds strongly to MT, the binding affinity of the head to the MT is very large so that the dissociation of the dimer from the MT is negligibly small. This implies that only when both heads are simultaneously in the ADP state is the dissociation of the dimer taken into account. In the calculations, when both heads move to positions of *y *>* *10 nm, the dimer is considered to dissociate from MT.

From 1000 simulated traces of the displacement of the center of mass of the dimer *versus* time, the total displacement in each trace can be obtained and the velocity in each trace can be calculated from the total displacement divided by the total time before dissociation. The run length and mean velocity are computed from all of individual displacements and velocities, respectively.

### The choice of parameter values

In this work, we focus on two types of kinesin‐1: *Drosophila* kinesin‐1 and CL human kinesin‐1. Values of the parameters related to the NL docking, the interaction between the kinesin head and MT, and the interaction between the two kinesin heads are taken as follows. We take the NL‐docking energy *E*_NL_ = 6*k*_B_ *T* for *Drosophila* kinesin‐1 and *E*_NL_ = 4.5*k*_B_ *T* for CL human kinesin‐1 (Table 1), as done before 38. Note that the residue mutation in the NL domain of CL human kinesin‐1 reduces *E*_NL_. We take *E*_w1_ = 19.8*k*_B_ *T* and *E*_w2_ = 40*k*_B_ *T* for *Drosophila* kinesin‐1, and *E*_w1_ = 25.3*k*_B_ *T* and *E*_w2_ = 43*k*_B_ *T* for CL human kinesin‐1 (Table 1). These values of *E*_w1_ = 19.8*k*_B_ *T* or 25.3*k*_B_ *T* and *E*_w2_ = 40*k*_B_ *T* or 43*k*_B_ *T* are close to those obtained by all‐atom MD simulations (X‐X. Shi, Y‐B. Fu, S‐K. Guo, P‐Y. Wang, H. Chen, P. Xie, under review), and different types could have slightly different values of the binding affinity of the head to MT. For both types of the kinesin, we take *t*_r_ = 10 μs (Table 1), as done before 37, 38. Values of the rate constant of NL docking, *k*_NL_, in different nucleotide states for both types of the kinesin are taken as follows. As discussed in detail in the Discussion section, the available experimental data indicated that the rate constant of the NL docking in the ADP.P_i_ state is much higher than that in the ATP state 47, 52, 53, 54. Thus, in the intermediate state, when the MT‐bound head is in the ADP.P_i_ state, we take *k*_NL_ = 800 s^−1^, which is consistent with the available biochemical data 55, while when the MT‐bound head is in ATP state, we take the rate constant of the NL docking to be very small, for example, *k*_NL_ = 1 s^−1^ in the calculation (Table 1). When the MT‐bound head is in the nucleotide‐free or ADP state, since its NL is unable to dock, we take *k*_NL_ = 0. Provided that *E*_I1_ > 40*k*_B_ *T* and *E*_I2_ < 20*k*_B_ *T*, varying the values of *E*_I1_ and *E*_I2_ has little effect on our results presented in this work.

**Table 1:** Values of parameters for WT *Drosophila* kinesin‐1 (DmK) and CL human kinesin‐1 (HsK)

Parameter	Value	
	DmK	HsK
*E*_NL_	6*k*_B_ *T*	4.5*k*_B_ *T*
*E*_w1_	19.8*k*_B_ *T*	25.3*k*_B_ *T*
*E*_w2_	40*k*_B_ *T*	43*k*_B_ *T*
*t*_r_ (μs)	10	10
*k*_NL_ (s^−1^) (ADP.P_i_ state)	800	800
*k*_NL_ (s^−1^) (ATP state)	1	1
*k*_c_ (s^−1^) (NL pointing forward)	185	185
$k_{c}^{\left(lead\right)}$ (s^−1^) (NL not pointing forward)	*k*_c_/40	*k*_c_/40
$k_{b}^{\left(T\right)}$ (μm ^−1^·s^−1^)	4	4
*k*_H_ (s^−1^)	350	350
*k*_–D_ (s^−1^)	370	370

The parameters related to ATPase activity are described as follows. The experimental data of Yildiz *et al*. 24 showed that extending the NLs by any length (implying that the tension on the NL is varied) has an insensitive effect on the ATPase rate of the dimer. On the other hand, the biochemical data showed that during the processive movement under saturating ATP, the rate‐limiting step of the ATPase activity is P_i_ release 56. Moreover, during a forward step, the P_i_ release in the trailing head can occur simultaneously with the ADP release, ATP binding, and then ATP hydrolysis in the leading head. Thus, we consider that the tension on the NL has no effect on the rate constant of P_i_ release; that is, the rate constant of P_i_ release is independent of the external load acting on the bead attached to the coiled‐coil stalk of the dimer, which is also consistent with the recent single‐molecule data 57. However, we consider that the rate constant of P_i_ release is dependent on the pointing direction of its NL. When the NL points in the forward direction, the head has a much larger rate constant of P_i_ release than when the NL points in the backward direction, which can be understood by considering that when pointing forward, the NL can interact with the head and the interaction enhances P_i_ release rate. This is consistent with the experimental data showing that by deleting the NL (implying that the interaction of the NL with the head is removed), the ATPase rate is reduced greatly, while the ADP release rate is unaffected 58. Thus, in the state with two heads bound simultaneously to MT, for the leading head, its rate constant of P_i_ release, $k_{c}^{\left(\mathrm{lead}\right)}$, is much smaller than that, $k_{c}^{\left(\mathrm{trail}\right)}\left(k_{c}^{\left(\mathrm{trail}\right)}=k_{c}\right)$, for the trailing head. In the intermediate state, when the NL of the MT‐bound head is stretched forward by a length of *l*_NL_ > 2.8 nm, it is considered to point forwards, and thus, the rate constant of P_i_ release is equal to *k*_c_, while in other cases, the rate constant of P_i_ release is equal to $k_{c}^{\left(\mathrm{lead}\right)}$. In the calculation, we take $k_{c}^{\left(\mathrm{lead}\right)}$ = *k*_c_/40 (Table 1), as done before 37, 38, and take *k*_c_ = 185 s^−1^ for both types of the kinesin (Table 1). This value of *k*_c_ is close to the biochemical data 5.

The second‐order rate constant, $k_{b}^{\left(T\right)}$, for ATP binding always has a constant value independent of the tension on and pointing direction of the NL, and we take $k_{b}^{\left(T\right)}$ = 4 μm ^−1^·s^−1^ for both types of the kinesin (Table 1), which is close to the biochemical data 5. The rate constant of ATP hydrolysis also always has a constant value independent of the tension on and pointing direction of the NL, and we take *k*_H_ = 350 s^−1^ for both types of the kinesin (Table 1), which is also consistent with the biochemical data 5.

Based on the available single‐molecule optical trapping data showing that the external force affects the affinity of ADP for kinesin, with the affinity under a backward force being smaller than under a forward force 59, we consider that the tension on the NL affects the rate constant of ADP release. Thus, we take the rate constant of MT‐stimulated ADP release from the trailing head, $k_{-D}^{\left(\mathrm{trail}\right)}=k_{-D}^{\left(\mathrm{lead}\right)}/\sigma \left(k_{-D}^{\left(\mathrm{lead}\right)}=k_{-D}\right)$, where σ is a constant larger than 1. In the intermediate state with MT‐bound head in the ADP state, the rate constant of MT‐stimulated ADP release is also taken to be $k_{-D}/\sigma$. To be consistent with the biochemical data 5, 56, we take *k*_–D_ **= 370 s^−1^ for both types of the kinesin (Table 1). In addition, we take σ = 3.1 for *Drosophila* kinesin‐1 and σ = 1.8 for CL human kinesin‐1. When ADP–head is detached from the MT, without MT stimulation, the ADP‐release rate is taken to be 0.

## Results

### Run length for wild‐type *Drosophila* kinesin‐1

**Figure 2:**
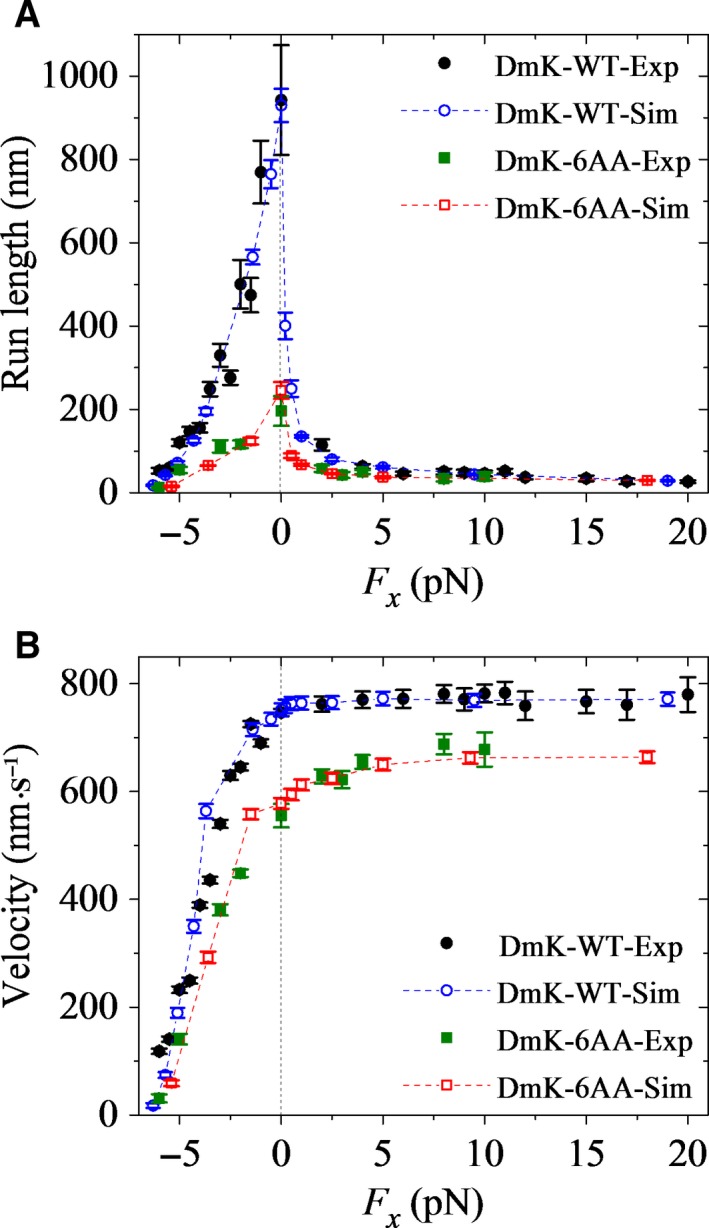
Dependence of run length and velocity of *Drosophila* kinesin‐1 upon the external force acting on the coiled‐coil stalk at saturating ATP. Open symbols are calculated data and filled symbols are experimental data taken from Milic *et al*. 22 and Andreasson *et al*. 23. Error bars represent SEM (*n *=* *1000). (A) Run length *versus* longitudinal component (*F*_*x*_) of the external force for DmK‐WT (circles) and DmK‐6AA (squares). (B) Velocity *versus* longitudinal component (*F*_*x*_) of the external force for DmK‐WT (circles) and DmK‐6AA (squares).

In this section, we study the effect of the external force on the run length of wild‐type *Drosophila* kinesin‐1 (called DmK‐WT), with the force–extension relation of the NL of one head obtained by all‐atom MD simulations being shown in Fig. S1. In Fig. 2A, we show our calculated results (open circles) of the run length *versus* the external force and compare with the experimental data (filled circles) of Milic *et al*. 22 and Andreasson *et al*. 23. The corresponding results for the velocity are shown in Fig. 2B. Here, the external force is defined as being positive and negative when it assists and resists the forward movement, respectively. It is seen that our calculated data for both the run length and velocity are in quantitative agreement with the experimental data. The data in Fig. 2A show a dramatic asymmetry of the run length with respect to the direction of the external force, with the run length under a moderate forward load being much shorter than that under no or a moderate backward load. These results can be understood as follows.

Since upon P_i_ release, the trailing head moves rapidly to the intermediate position, and after the NL docking of the MT‐bound head, the tethered ADP–head moves rapidly to the next forward MT‐binding site or to the previous backward MT‐binding site, the detaching of the dimer from MT occurs mainly in the intermediate state, where one head is bound to MT, with its NL being undocked, and the other ADP–head is bound strongly to the MT‐bound head. Furthermore, since in nucleotide‐free, ATP, or ADP.P_i_ state, the MT‐bound head binds strongly to MT, its dissociation from MT is negligible. Thus, the detaching of the dimer from MT occurs mainly during two periods in the intermediate state. One period (called Period I) is after P_i_ release occurs in the MT‐bound head and before the affinity of the MT‐bound ADP–head for the local MT changes from *E*_w1_ to *E*_w2_ (Fig. 1G). The other period (called Period II) is when the MT‐bound head is in the ADP state with its affinity for MT being *E*_w2_ (Fig. 1H and I). In Period I, upon P_i_ release, the binding affinity of the MT‐bound head to the local MT becomes very weak, with a small affinity *E*_w1_; the head has a very large probability to detach from MT within time *t*_r_. In Period II, since ADP–head binds to MT weakly, with an affinity *E*_w2_, the head also has a large probability to detach from MT before ADP release within a time (1/*k*_–D_) that is much longer than *t*_r_.

That the run length under a moderate forward load is much shorter than that under no or a moderate backward load arises from the detaching of dimer from MT that occurs in Period I. In the intermediate state, before the NL of the MT‐bound head is docked, the NL is driven to point in the forward direction under the forward load, and by contrast, it is not pointed in the forward direction under no or the backward load. Thus, in the intermediate state, before the NL docking, the rate constant of P_i_ release from the MT‐bound head under the forward load is much larger than that under no or the backward load (see the section ‘The choice of parameter values’ in Materials and methods). The result is that in the intermediate state, before the NL docking, the probability of P_i_ release occurring in the MT‐bound head, that is, the occurrence probability of Period I, under the forward load is significantly larger than that under no or the backward load. Consequently, the dimer at the intermediate state has a much larger probability to detach from MT under the forward load than under no or the moderate backward load.

**Figure 3:**
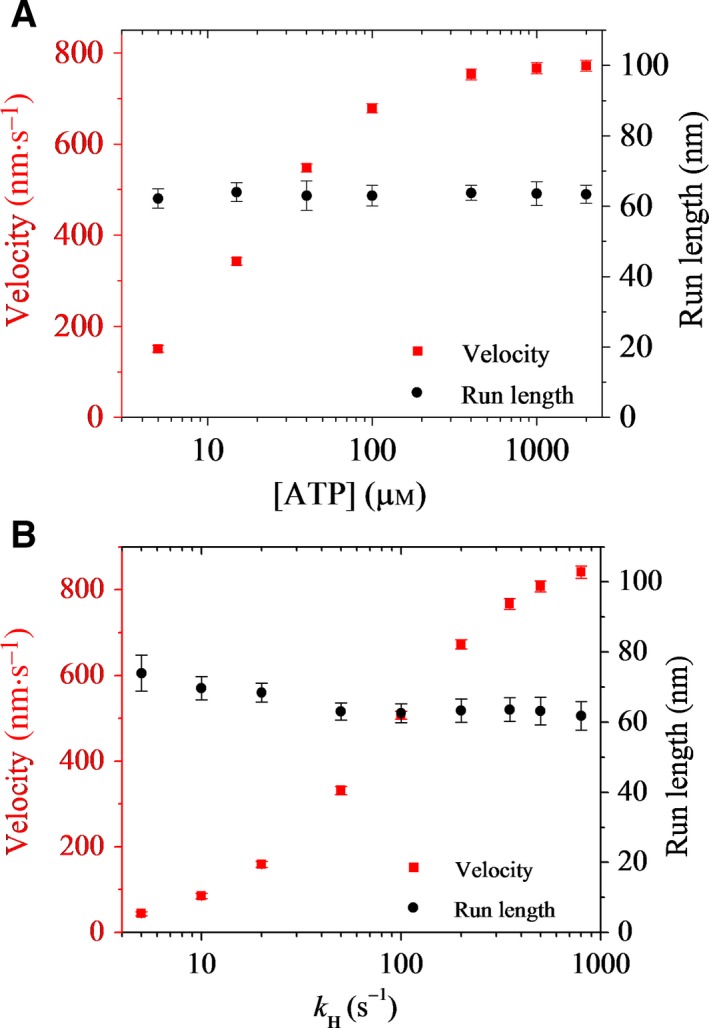
Effects of ATP concentration and replacing ATP with a slowly hydrolyzable ATP analog, ATPγS, on run length and velocity of *Drosophila* kinesin‐1. Error bars represent SEM (*n *= 1000). (A) Run length (circles) and velocity (squares) *versus* ATP concentration under a given longitudinal component of the external force acting on the coiled‐coil stalk, *F*_*x*_ = +4 pN. (B) Run length (circles) and velocity (squares) *versus* rate constant of ATPγS hydrolysis, *k*_H_, under *F*_*x*_ = +4 pN and saturating ATPγS.

In the above (Fig. 2), we focus on the case at saturating ATP. Now, we consider the case at low ATP concentrations. In Fig. 3A, we show the calculated results of the run length *versus* [ATP] at a given longitudinal component of the external force, *F*_*x*_ = +4 pN (filled circles). The corresponding results of velocity *versus* [ATP] are also shown in Fig. 3A (filled squares). It is seen that although the velocity increases sensitively with the increase of [ATP], the run length is nearly independent of [ATP], which can be explained as follows. Before ATP binding, the dimer is most of the time in the state with the nucleotide‐free head bound strongly to MT and the detached ADP–head in the intermediate position bound strongly to the nucleotide‐free head, which is consistent with the experimental and structural observations 60, 61. Since the nucleotide‐free head binds strongly to MT, it is considered not to dissociate from MT. After ATP binding, since ATP hydrolysis is rapid and the NL docking is very slow, the NL is almost unable to dock before ATP hydrolysis to ADP.P_i_. Hence, it is expected that the dissociation probability of the dimer would be nearly the same as that for the case at saturating [ATP], giving the run length at a very low [ATP] to be nearly the same as that at saturating [ATP]. In our calculations, for simplicity, we have neglected the dissociation during the period when the kinesin head in the nucleotide‐free state binds strongly to MT. Considering the small dissociation probability during the *long* period in the nucleotide‐free state of the MT‐bound head, the run length at low [ATP] would become slightly smaller than that at saturating [ATP], which is in good agreement with the single‐molecule data of Milic *et al*. 22 and Andreasson *et al*. 23.

We next consider the case at saturating ATPγS, a slowly hydrolyzable ATP analog 22. For this case, the dimer is most of the time in the state with two heads in the ATPγS or ADP.γSPi state binding strongly to MT, with no dissociation. After γSPi is released in the trailing head, the tethered ADP–head moves rapidly to the intermediate position. Since in the intermediate state the MT‐bound head is most probably in the ADP.γSPi or ATPγS state, and in the ATPγS state the NL docking is very slow, it is expected that the dissociation probability of the dimer would be similar to that for the case with ATP, giving a similar run length for the two cases. For example, in Fig. 3B, we show the calculated results of run length *versus* the rate constant of ATPγS hydrolysis, *k*_H_, under *F*_*x*_ = +4 pN (filled circles), with values of other parameters being unchanged. The corresponding results for the velocity are also shown in Fig. 3B (filled squares). It is seen that although the velocity increases sensitively with the increase in *k*_H_, the run length decreases only slightly with the increase in *k*_H_. In our calculations, for simplicity, we have neglected the dissociation during the period when the kinesin head in ATPγS or ADP.γSPi state binds strongly to MT. Considering the small dissociation probability during the *long* period in the ATPγS state, the run length at low *k*_H_ would become closer to that for the case with ATP (with *k*_H_ = 350 s^−1^), implying that the run length for the case with ATPγS is close to that for the case with ATP. These are in good agreement with the single‐molecule data of Milic *et al*. 22 and Andreasson *et al*. 23.

### Run length for *Drosophila* kinesin‐1 with extended neck linkers

In this section, we study the effect of the external force on the run length of *Drosophila* kinesin‐1 with extended NLs. As calculated in our previous work 38, for kinesin‐1 dimers with one, two, three, four, five, and six additional amino acid residues inserted into the C‐terminal portion of the linker region of each head, their velocities across all negative loads only have small changes but have large drops relative to those with no extension of the NLs, which is consistent with the experimental data 23. Here, we only focus on the kinesin with six additional amino acid residues (with sequence LQASQT) inserted into the C‐terminal portion of the linker region of each head (called DmK‐6AA), as done in Andreasson *et al*. 23. Since the amino acid residues inserted into the NL are far away from the motor domain, the insertions should have no effect on the interaction of the head with MT, the interaction between the two heads and the NL docking. As the available experimental data indicated 24, the insertions have no effect on the rate constants of ATP hydrolysis and P_i_ release. However, since the single‐molecule data indicated that the tension on the NL affects the rate constant of ADP release 59, the insertions should affect the rate constant of MT‐stimulated ADP release. Consequently, we take the same values for all parameters for DmK‐6AA as those for DmK‐WT except *k*_–D_ and the force–extension relation of the NL. Using all‐atom MD simulations, the calculated results of the force–extension relation of the NL of one head for DmK‐6AA are shown in Fig. S2. From Figs S1 and S2, it is seen that when the two heads are bound to MT simultaneously, with one NL being stretched to a length of *d*/2 = 4.1 nm, the internally stretched force between the two heads is nearly zero for DmK‐6AA, whereas it is about 30 pN for DmK‐WT. Thus, in the calculations, we take the rate constant of ADP release from both the leading and trailing heads for DmK‐6AA to be $k_{-D}/\sigma$ (σ = 3.1) (see section ‘The choice of parameter values’ in Materials and methods). In Fig. 2A, we also show our calculated results (open squares) of the run length for DmK‐6AA *versus* the external force and compare with the experimental data (filled squares) of Andreasson *et al*. 23. The corresponding results for the velocity are shown in Fig. 2B. It is seen that the calculated data for both run length and velocity are also in good agreement with the experimental data. That the origin of the run length of DmK‐6AA under no or a backward load is greatly smaller than that of DmK‐WT is explained as follows.

For *Drosophila* kinesin‐1, the value of *E*_w2_ = 40*k*_B_ *T* (see Table 1) gives a larger probability of the dimer detaching from MT during Period II than during Period I under no or a backward load. On the other hand, for DmK‐6AA, the rate constant of ADP release from the leading head is slower than that for DmK‐WT, leading to the occurrence probability of Period II for DmK‐6AA being larger than that for DmK‐WT. Consequently, the probability of the dimer detaching from MT for DmK‐6AA is larger than that for DmK‐WT, leading to the run length of the former being smaller than that of the latter. By contrast, under a forward load, the run length of DmK‐6AA is close to that for DmK‐WT (Fig. 2A). This is because under the forward load, the run length is mainly determined by the detaching of dimer from MT that occurs in Period I (see above), and for both DmK‐6AA and DmK‐WT, the occurrence probabilities of Period I are comparable.

### Durations of one‐head‐bound and two‐head‐bound states

Recently, using high‐resolution single‐molecule microscopy, Mickolajczyk and Hancock 62 observed that for *Drosophila* kinesin‐1, extending the NLs increases the mean duration of the 1HB state, while it has little effect on the mean duration of the 2HB state in one step of processive movement of the dimer. To explain these data, we calculate the mean duration of 1HB state to be about 3.2 ms for DmK‐WT and to be about 6.1 ms for DmK‐6AA under no load and saturating ATP (2 mm), with the latter being evidently larger than the former, and the mean duration of the 2HB state to be about 7.8 ms for DmK‐WT and about 8.1 ms for DmK‐6AA, with the two values being close to each other. These are in agreement with the single‐molecule data 62. The origin of the above feature is explained as follows. Since the rate constant of ADP release in the leading head for DmK‐6AA is slower than that for DmK‐WT, there is a larger probability for DmK‐6AA to be in the 1HB state with MT‐bound head bound by ADP than for DmK‐WT. On the other hand, the duration of the 1HB state is equal to the total time of ADP release, ATP binding, ATP hydrolysis, and then NL docking in the MT‐bound head, with the rate constants of ATP binding (*k*_b_), ATP hydrolysis (*k*_H_), and NL docking (*k*_NL_) being the same for both DmK‐6AA and DmK‐WT. Thus, it is expected that the mean duration of the 1HB state for DmK‐6AA is larger than for DmK‐WT. By contrast, based on our model, the duration of the 2HB state is mainly determined by the time of P_i_ release in the trailing head. Since P_i_ release rate is independent of the NL length, it is expected that the mean duration of the 2HB state for DmK‐6AA is close to that for DmK‐WT.

In addition, the biochemical data of Mickolajczyk and Hancock 62 showed that extending the NLs of *Drosophila* kinesin‐1 increases the time of half‐site ADP release at saturating ATP. In our model, the time of half‐site ADP release at saturating ATP is approximately calculated by τ_half_ = 1/*k*_H_ + 1/*k*_c_ + 1/*k*_NL_ + 1/*k*_–D_. Since terms *k*_H_, *k*_c_, and *k*_NL_ are the same for both DmK‐6AA and DmK‐WT, whereas *k*_–D_ (the rate constant of ADP release in the leading head) for DmK‐6AA is smaller than that for DmK‐WT, it is expected that τ_half_ for DmK‐6AA is larger than that for DmK‐WT. The calculated value of τ_half_ is about 17.89 ms for DmK‐6AA and is about 12.22 ms for DmK‐WT, with the difference Δτ_half_ = 5.67 ms, in good agreement with the biochemical data 62.

**Figure 4:**
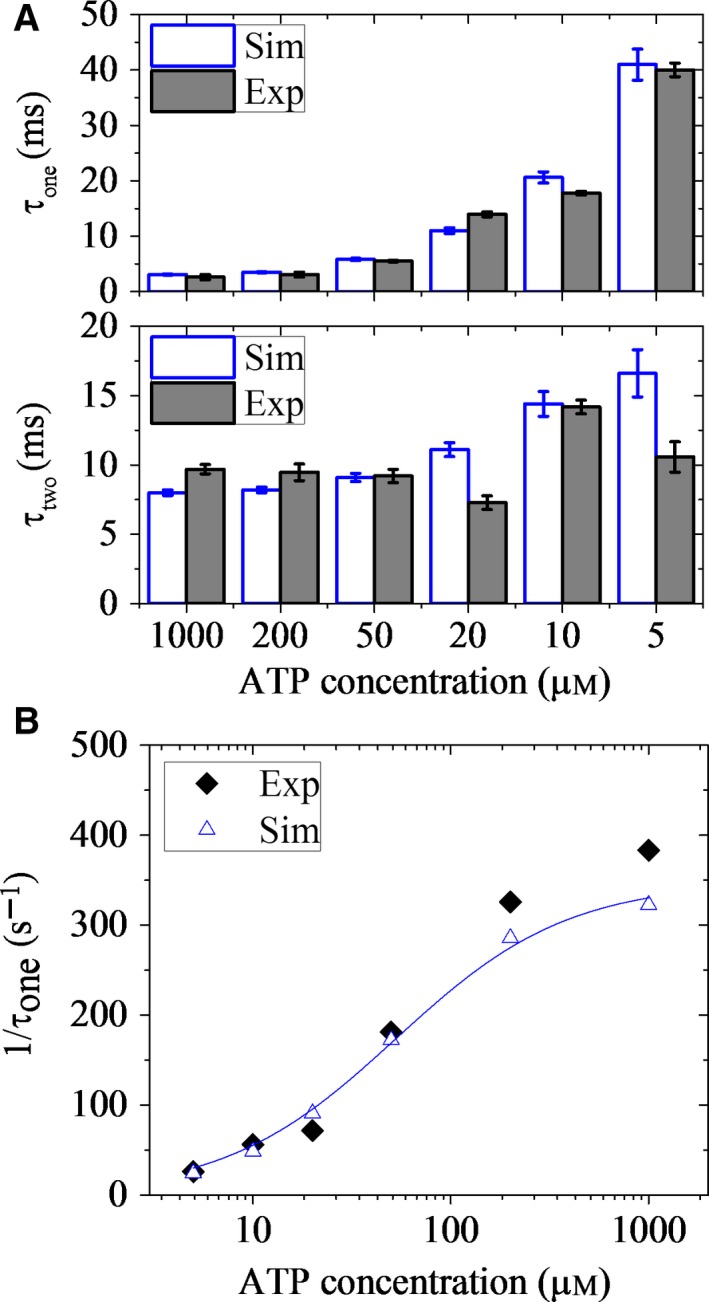
Durations of one‐head‐bound (1HB) and two‐head‐bound (2HB) states during processive movement of cysteine‐light (CL) human kinesin‐1 under no load. (A) Mean durations of 1HB state (upper) and 2HB state (lower) *versus* ATP concentration. Open columns are calculated data, and filled columns are experimental data taken from Isojima *et al*. 60. Error bars represent SEM (*n *= 1000). (B) Inverse of the mean duration of 1HB state as a function of ATP concentration. Open symbols are calculated data, and filled symbols are experimental data taken from Isojima *et al*. 60. Line is the fit with the Michaelis–Menten equation, 1/τ_1_ = *k*_cat_ [ATP]/*K*_M__* *_+ [ATP]). The fit parameters are *k*_cat _= 348 s^−1^ and *K*_M_ = 53 μm.

Moreover, we studied the mean durations of the 1HB and 2HB states *versus* [ATP] for CL human kinesin‐1, with the calculated results being shown in Fig. 4A, where the single‐molecule data of Isojima *et al*. 60 on CL human kinesin‐1 are also shown for comparison. It is seen that the mean duration of the 1HB state increases significantly with the decrease in [ATP], whereas the mean duration of the 2HB state increases only slightly with the decrease in [ATP]. These results are in quantitative agreement with the experimental data. The inverse of the mean duration of the 1HB state *versus* [ATP] satisfies the Michaelis–Menten relation (Fig. 4B), which is also in agreement with the single‐molecule data of Isojima *et al*. 60.

### Run length for cysteine‐light human kinesin‐1

As seen above (Fig. 2A), for *Drosophila* kinesin‐1, under no load, the run length of DmK‐6AA is reduced greatly compared with that of DmK‐WT. By contrast, for CL human kinesin‐1, the available single‐molecule data showed that the extension of NLs has an insensitive effect on or even increases slightly the run length under no load although it has a sensitive effect on or decreases largely the velocity 24, 25. Here, we give quantitative explanations of these contrary results.

**Figure 5:**
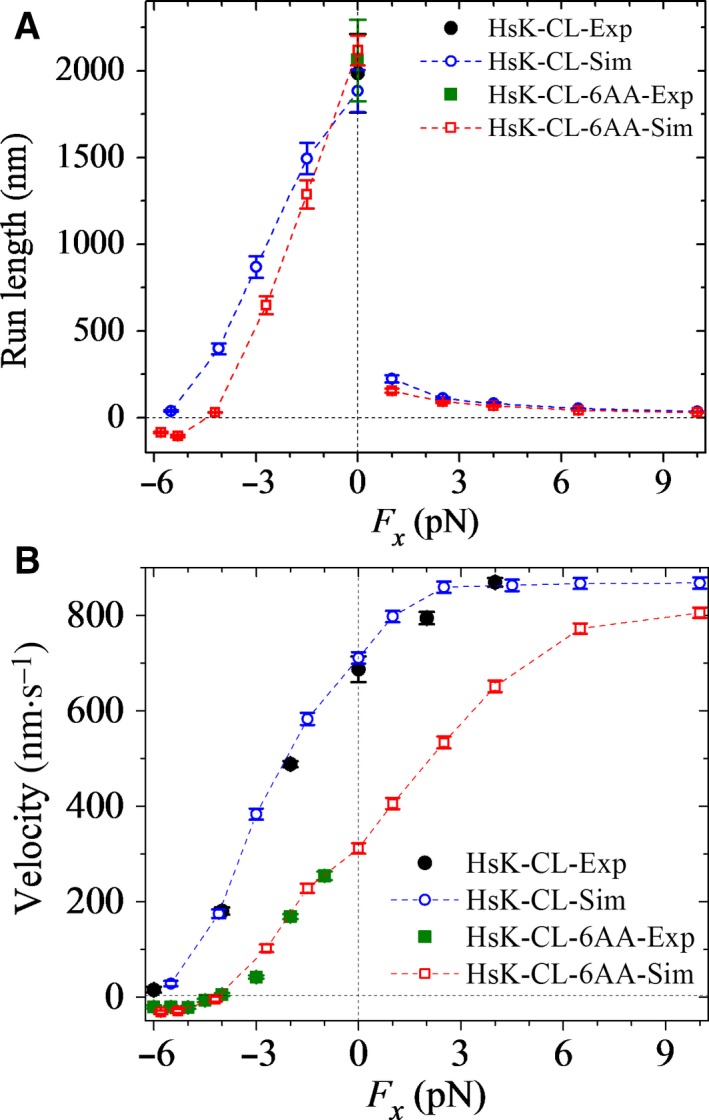
Dependence of run length and velocity of cysteine‐light (CL) human kinesin‐1 upon the external force acting on the coiled‐coil stalk at saturating ATP. Open symbols are calculated data. Error bars represent SEM (*n *=* *1000). (A) Run length *versus* longitudinal component (*F*_*x*_) of the external force for HsK‐CL (circles) and HsK‐CL‐6AA (squares). Filled symbols are experimental data taken from Yildiz *et al*. 24. (B) Velocity *versus* longitudinal component (*F*_*x*_) of the external force for HsK‐CL (circles) and HsK‐CL‐6AA (squares). Filled symbols are experimental data taken from Andreasson *et al*. 23 and Clancy *et al*. 25.

First, we studied the effect of the external force on the run length of CL human kinesin‐1 without extension of the NLs (called HsK‐CL), with the force–extension relation of the NL of one head obtained by all‐atom MD simulations being shown in Fig. S3. The calculated results of the run length *versus* the external force are shown in Fig. 5A (open circles), where for comparison, the available experimental value of the run length under no load (filled circle) 24 is also shown. The corresponding results for the velocity are shown in Fig. 5B. It is seen that the calculated data for both the run length and velocity are in quantitative agreement with the experimental data. As expected, the curve of the run length *versus* the external load for HsK‐CL (Fig. 5A) resembles that for DmK‐WT (Fig. 2A), both showing the dramatic asymmetry with respect to the direction of the load.

Then, we studied the effect of the external force on the run length of CL human kinesin‐1 with extended NLs. Here, we focus on the CL human kinesin‐1 with six additional amino acid residues (e.g. with sequence AEQKLT) inserted into the C‐terminal portion of the linker region of each head (called HsK‐CL‐6AA). Our calculated data of both the run length and velocity are insensitive to the sequences of the inserted amino acids. As mentioned above for DmK‐6AA, we take the same values for all parameters for HsK‐CL‐6AA as those for HsK‐CL except *k*_–D_ and the force–extension relation of the NL. As mentioned above for DmK‐6AA, we take the rate constant of ADP release from both the leading and trailing heads for HsK‐CL‐6AA to be *k*_–D_/σ (σ = 1.8) (see ‘The choice of parameter values’ in Materials and methods). The results of the force–extension relation of the NL of one head for HsK‐CL‐6AA calculated using all‐atom MD simulations are shown in Fig. S4. In Fig. 5A, we also show the calculated results of the run length *versus* the external force for HsK‐CL‐6AA (open squares), where for comparison, the available experimental value of the run length under no load (filled square) 24 is also shown. The corresponding results for the velocity are shown in Fig. 5B. It is seen that the calculated data for both the run length and velocity are in quantitative agreement with the experimental data. More puzzling, it is noted that the run length of HsK‐CL‐6AA under no load is slightly larger than that of HsK‐CL (Fig. 5A), and by contrast, the run length of DmK‐6AA under no load is much smaller than that of DmK‐WT (Fig. 2A). The contrary features between HsK‐CL‐6AA and DmK‐6AA compared with those with no extension of the NLs are explained as follows.

As mentioned in the above section, the value of *E*_w2_ = 40*k*_B_ *T* for *Drosophila* kinesin‐1 gives the detaching of the dimer from MT arising mainly from that occurring during Period II under no load, resulting in the probability of the dimer detaching from MT for DmK‐6AA being larger than that for DmK‐WT. By contrast, the value of *E*_w2_ = 43*k*_B_ *T* (see Table 1) for CL human kinesin‐1 gives the detaching of the dimer from MT as arising mainly from that occurring during Period I and to a minor extent from that occurring during Period II under no load. Since for HsK‐CL‐6AA, the rate constant of ADP release from the leading head is smaller than that for HsK‐CL, the ensuing ATP binding, ATP hydrolysis, and then P_i_ release in the leading or MT‐bound head are delayed for HsK‐CL‐6AA relative to those for HsK‐CL. As a result, under no load, the probability for P_i_ release to occur before NL docking at the intermediate state for HsK‐CL‐6AA is smaller than for HsK‐CL; that is, the probability for Period I to occur for HsK‐CL‐6AA is smaller than for HsK‐CL. Thus, although the probability for Period II to occur for HsK‐CL‐6AA is larger than for HsK‐CL, considering that the detaching during Period II makes a minor contribution it is understandable that the total probability of the dimer detaching from MT in one step for HsK‐CL‐6AA is slightly smaller than that for HsK‐CL. As a result, the run length of the former is slightly larger than that of the latter.

Furthermore, it is interesting to see from Figs 2B and 5B that compared to the case with no extension of the NLs, the velocity of HsK‐CL‐6AA under no load has a larger reduction than DmK‐6AA. By contrast, as shown in Figs 2A and 5A, the run length of HsK‐CL‐6AA under no load is slightly larger than that with no extension of the NLs, whereas the run length of DmK‐6AA is reduced greatly. More interestingly, it is seen from Figs 2B and 5B that even under a large forward load (20 pN), the velocity of DmK‐6AA is evidently smaller than DmK‐WT, and by contrast, only under a moderate forward load (e.g. 9 pN), the velocity of HsK‐CL‐6AA approaches nearly to that of HsK‐CL, which is in agreement with the single‐molecule data of Yildiz *et al*. 24. Additionally, it is seen from Fig. 5 that HsK‐CL‐6AA is capable of undertaking many rearward steps when –6 pN ≤ *F*_*x*_ ≤ –5 pN, with negative velocity and run length, whereas no such processive backstepping is observed for HsK‐CL when *F*_*x*_ ≥ –6 pN, with the velocity and run length having positive values. By contrast, from Fig. 2B, it is seen that both DmK‐6AA and DmK‐WT show no such processive backstepping when *F*_*x*_ ≥ –6 pN, with positive velocity and run length.

### Effect of the presence of additional P_i_ in solution on run length

**Figure 6:**
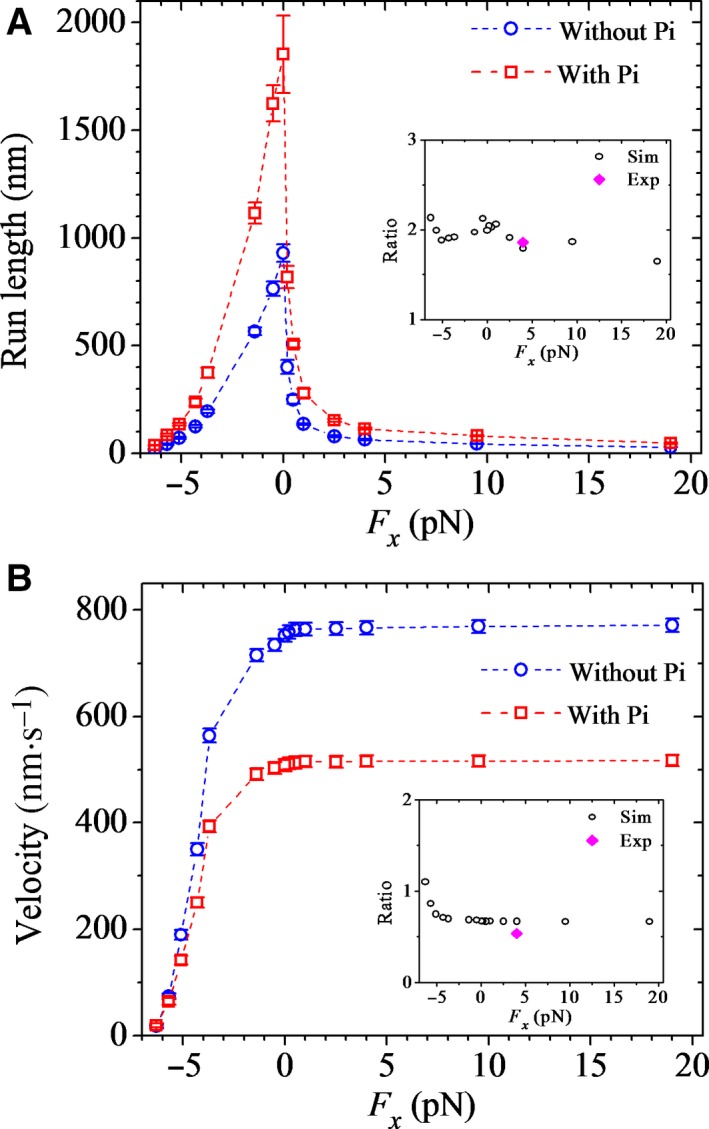
Effects of the presence of additional P_i_ in solution on run length and velocity of *Drosophila* kinesin‐1 at saturating ATP. The results are calculated with γ = 2.3. Error bars represent SEM (*n *=* *1000). (A) Run length *versus* longitudinal component (*F*_*x*_) of the external force acting on the coiled‐coil stalk for DmK‐WT. Inset shows ratios of the run length in the presence of additional P_i_ in solution to the corresponding one in the absence of additional P_i_ (open symbols), and the ratio of the experimental data at [P_i_] = 100 mm to that at [P_i_] = 0 under the forward external force of 4 pN (filled symbol) is also shown 22. (B) Velocity *versus* longitudinal component (*F*_*x*_) of the external force for DmK‐WT. Inset shows ratios of the velocity in the presence of additional P_i_ in solution to the corresponding one in the absence of additional P_i_ (open symbols), and the ratio of the experimental data at [P_i_] = 100 mm to that at [P_i_] = 0 under the forward external force of 4 pN (filled symbol) is also shown 22.

For simplicity of analysis, we consider that the presence of additional P_i_ in solution increases the lifetime of the post‐hydrolysis ADP.P_i_ state, as done in Milic *et al*. 22, which can be interpreted as inhibition of P_i_ release. In the calculation, this consideration is equivalent to the reduction in rate constant of P_i_ release, *k*_c_. Thus, we calculate the run length and velocity for DmK‐WT by taking the value of *k*_c_ to be decreased by γ‐fold (γ > 1) compared to that used in Fig. 2 and values of other parameters to be the same as those used in Fig. 2. In Fig. 6A,B, we show the calculated results of the run length and velocity, respectively, *versus* external force with γ = 2.3. For comparisons, the calculated data of Fig. 2 without additional P_i_ in solution are also reshown in Fig. 6. In insets of Fig. 6, we show the ratios of the calculated run length and velocity in the presence of additional P_i_ to the corresponding ones without the additional P_i_, where for comparison the ratios of available single‐molecule data with 100 mm P_i_ in solution to the corresponding ones without additional P_i_ in solution at *F*_*x*_ = 4 pN 22 are also shown. The ratios of the calculated run length and velocity in the presence of additional P_i_ to the corresponding ones without the additional P_i_ at *F*_*x*_ = 4 pN with varying γ are shown in Fig. S5. From Figs 6 and S5, it is seen that with γ = 2.3, the calculated data are in agreement with the single‐molecule data. Moreover, from Figs 6 and S5, we see that the presence of additional P_i_ increases the run length, which is in agreement with the single‐molecule data 22, and by contrast, the presence of additional P_i_ decreases the velocity, which is also consistent with the single‐molecule data 22. These are easily understandable because decreasing *k*_c_ reduces the occurrence probabilities of both Period I and Period II, which results in the reduction in the dissociation probability of the dimer, and decreasing *k*_c_ reduces evidently the velocity.

### Dependence of run length on external force acting on one head

In this section, we study the run length and velocity of wild‐type *Drosophila* kinesin‐1 with one head being attached to a micrometer‐sized bead and an external load acting on the bead, as done in the single‐molecule optical trapping assays of Guydosh and Block 63, explaining the experimental data on the velocity 63 and providing predicted results on the run length. For this case, the equations for the mechanical stepping of the dimers are given in Section S6. By comparing the experimental data of Guydosh and Block 63 with those of Andreasson *et al*. 23, it is seen that the velocity under no load for the former is slightly smaller than that for the latter. Thus, to be consistent with the experimental data of Guydosh and Block 63, we take *k*_c_ = 120 s^−1^, which is smaller than the value given in Table 1, with other parameters having the same values as those given in Table 1.

**Figure 7:**
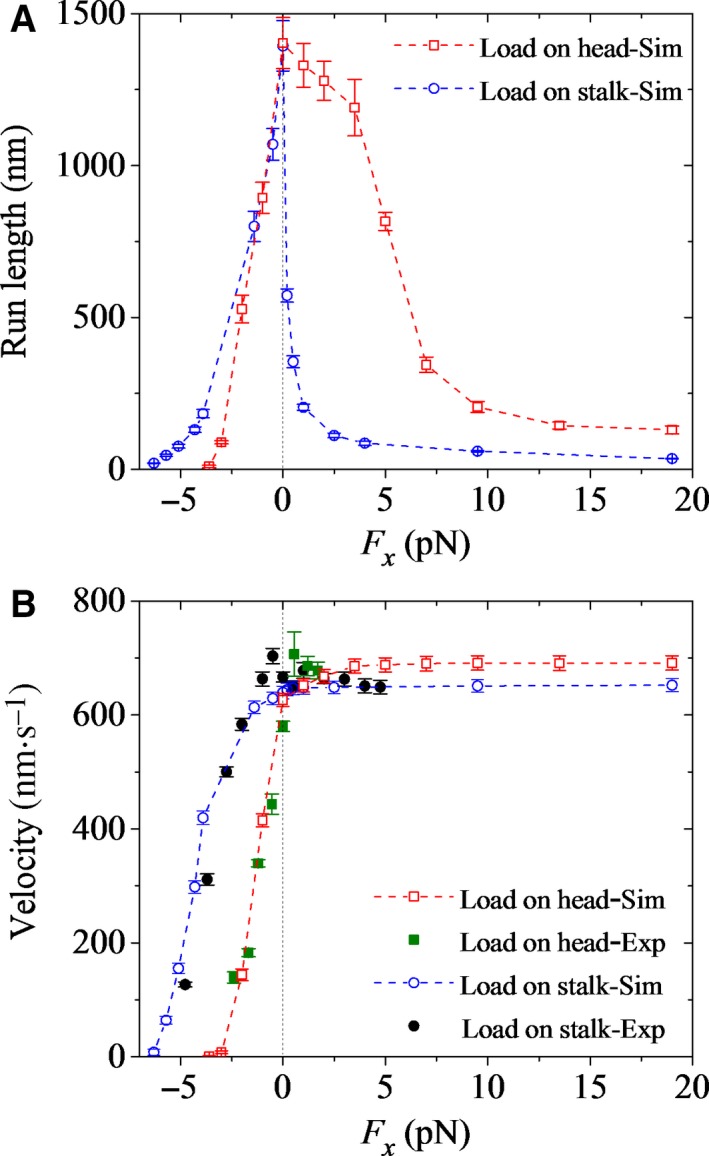
Dependence of run length and velocity of *Drosophila* kinesin‐1 upon the external force at saturating ATP. Error bars represent SEM (*n *= 1000). (A) Run length *versus* longitudinal component (*F*_*x*_) of the external force acting on one head (open squares). For comparisons, the calculated data for the case of the external force acting on the coiled‐coil stalk (open circles) are also shown. (B) Velocity *versus* longitudinal component (*F*_*x*_) of the external force acting on one head (open squares). For comparisons, the calculated data for the case of the external force acting on the coiled‐coil stalk (open circles) are also shown. Open symbols are calculated data, and filled symbols are experimental data taken from Guydosh and Block 63.

Figure 7A,B shows our calculated results (open squares) of the load dependences of run length and velocity, respectively, where the corresponding available experimental data on the velocity (filled squares) of Guydosh and Block 63 are also shown. For comparisons, in Fig. 7, we also show the calculated results (open circles) for the case under the external load acting on the bead attached to the coiled‐coil stalk and the corresponding experimental data on the velocity (filled circles) of Guydosh and Block 63. From Fig. 7B, we see that the calculated data on the velocity for both the case with the bead attached to one head and the case with the bead attached to the coiled‐coil stalk are in agreement with the experimental data. Additionally, it is seen that under the forward load, the velocity for the case with the bead attached to one head is slightly larger than that for the case with the bead attached to the coiled‐coil stalk. This is explained as follows. Under the forward load acting on the detached head, after it moves in front of the MT‐bound head, it is still acted on by the forward load, enhancing the probability for the detached head to move directly to the next forward MT‐binding site without staying at the intermediate position for a short time of 1/*k*_NL_ and thus bringing forward the time of ADP release. By contrast, under the forward load acting on the stalk, after the detached head moves in front of the MT‐bound head, the load acts on the MT‐bound head rather than on the detached head. Thus, the forward load has little effect on the probability for the detached head to move directly to the next forward MT‐binding site.

From Fig. 7A, we see that for the case with the bead attached to one head, the run length decreases sensitively with the increase in the magnitude of the backward load, which is similar to the case with the bead attached to the coiled‐coil stalk. This is easily understandable, because for both the cases, the decrease in the run length arises from both the decrease in the velocity and the increase in the rate constant of dissociation of the motor from MT that occurs during Period II. Interestingly, from Fig. 7A, we see that for the case with the bead attached to one head, the run length decreases mildly with the increase in the forward load, which is in contrast to the case with the bead attached to the coiled‐coil stalk, where the run length drops sharply under even a small forward load. In other words, for the former case, the dependence of run length on load is consistent with the expectation from Kramers theory, whereas for the latter case, the dependence of run length on load is contrary to the expectation from Kramers theory. This is because for the former case, the forward load has no effect on the pointing direction of the NL of the MT‐bound head in the intermediate state, whereas for the latter case, the forward load drives the NL of the MT‐bound head to point forward, greatly accelerating its P_i_ release and thus greatly increasing the occurrence probability of Period I. For the former case, the mild decrease in the run length with the increase in the forward load arises from the mild increase of the rate constant of dissociation of the motor from MT that occurs during Period II. By contrast, for the latter case, the sharp decrease in the run length under even a small forward load arises from the large probability of dissociation that occurs during Period I.

## Discussion

In the model presented previously 37, 38, it was assumed that when the MT‐bound head is in the ATP or ADP.P_i_ state, once the detached head is near the intermediate position, the NL of the MT‐bound head is docked into its motor domain immediately, implying that the NL docking occurs with a rate of *k*_NL_ *→* ∞. With this model, diverse experimental data on the dynamics of kinesin‐1 except the processivity were explained quantitatively 37, 38. In this work, to be consistent with the available fluorescence polarization microscopy data 47, we improve the model by assuming that in the intermediate state, *k*_NL_ has a small value when the MT‐bound head is in the ATP state and has a finite and large value when the MT‐bound head is in the ADP.P_i_ state to study the processivity of kinesin‐1. This implies that even at saturating ATP, there is a time period in which the detached head is in the intermediate position relative to the MT‐bound head before diffusing to the next MT‐binding site. This is consistent with the recent single‐molecule data of Isojima *et al*. 60 and Mickolajczyk *et al*. 52. In the intermediate state with the detached ADP–head bound strongly to the MT‐bound head, the N‐terminal strand β0 of the motor domain of the MT‐bound head and the strand β9 of the NL are in proximity so that the cover–neck bundle is able to form; that is, the NL is able to dock 48, 64. On the other hand, it takes time for the NL docking to complete. As a consequence, it is inferred that the existence of the intermediate state could function biologically to make the NL docking take place efficiently. The NL docking then prevents the tethered ADP–head from moving backward under the backward load after it moves to the position in front of the MT‐bound head, thus increasing the stall force 37 rather than acting as the driving force to make the ADP–head detach from the previous MT‐binding site and then move forward.

### Nucleotide‐dependent neck‐linker‐docking efficiency

Based on the fluorescence polarization microscopy data showing that in the ATP state, the NL has a lower docking efficiency than in the ADP.P_i_ state 47 and the single‐molecule optical trapping and microscopy data 22, 52, it has been proposed that in the ATP state, the NL is only partially docked, while in the ADP.P_i_ state, the NL docking is complete 22, 52. If this is true, it would imply that with the non‐hydrolyzable ATP analog AMP‐PNP, the dimer cannot transit from the 1HB state into the 2HB state, and only in the ADP.P_i_ state can the dimer enter into the 2HB state. However, many studies with AMP‐PNP have shown that hydrolysis is not required for the dimer to enter the 2HB state 43, 53, 65, 66, 67. Moreover, the experimental data showed that AMP‐PNP and ATPγS can also trigger release of ADP from a tethered intermediate, but with a much smaller rate than ADP.P_i_ 52, 53, 54. Thus, to be consistent with all of the experimental data, we propose here that in the ATP state the NL docking is less efficient, with a very slow rate, while in the ADP.P_i_ state, the NL docking is efficient, with a high rate.

### The model can explain diverse aspects of movement dynamics

Since the change of the binding affinity of ADP–head to the previous MT‐binding site from *E*_w1_ to *E*_w2_ takes a very short time, *t*_r_ = 10 μs, it is evident that the finite value of *k*_NL_ (with 1/*k*_NL_ = 1.25 ms in the ADP.P_i_ state) has little effect on all of the theoretical data for kinesin‐1 presented in the previous work 37, 38. For example, some typical data are shown in Figs 2B, 5B and 7B showing the effects of the external force and extension of NLs on the velocity, in Fig. S6 showing the effect of NL docking on the velocity, and in Fig. S7 showing the effect of solution viscosity on the velocity. Thus, the modified model presented in this work can not only quantitatively explain diverse experimental data on kinesin‐1 dynamics such as the velocity, mechanochemical coupling ratio, and limping effects, as done in the previous work 37, 38, but also explain quantitatively experimental data on the processivity 22, 23, 24.

### The sensitivity of the calculated results to the relevant parameters

As it is time consuming, in this work, we only made detailed calculations with parameter values (see Table 1) that can give good agreement with the available experimental data. It is instructive to see the sensitivity of the calculated results to the relevant parameters. For example, some results for DmK‐WT are given in Fig. S8, where we change one of its parameter values while keeping other parameter values unchanged.

From Fig. S8A, it is seen that changing *E*_NL_ by 0.6*k*_B_ *T* has only a slight (< 1%) effect on the velocity and run length under the forward and low backward loads. Under the large backward load (e.g. *F*_*x*_ = –4.3 pN), increasing and decreasing *E*_NL_ by 0.6*k*_B_ *T* increases and decreases the run length by about 1% and 13%, respectively, and increases and decreases the velocity by about 28% and 34%, respectively. This can be understood as follows. The energy barrier *E*_NL_ prevents the tethered ADP–head in the intermediate position from moving backward to the trailing position. Under the forward and low backward loads, this backward movement probability is very low, and thus changing *E*_NL_ by a small value has only a slight effect on the motility. Under the large backward load, this backward movement probability is high, and thus changing *E*_NL_ has a larger effect on the motility.

From Fig. S8B, it is seen that changing *E*_w1_ by 1*k*_B_ *T* has nearly no effect on both the run length and the velocity. This can be understood as follows. Under the internally stretched force of the NLs for DmK‐WT with both heads bound to MT, after P_i_ release occurs in the trailing head the head has a nearly 100% probability to escape the potential well of depth *E*_w1_ ≤ 20.8*k*_B_ *T* within time *t*_r_ 37. Thus, provided *E*_w1_ ≤ 20.8*k*_B_ *T*, the change of *E*_w1_ has nearly no effect on the motility of DmK‐WT. However, for DmK‐6AA with longer NL length, after P_i_ release occurs in the trailing head, the head does not have a 100% probability to escape the potential well of depth *E*_w1_ = 19.8 *k*_B_ *T* within time *t*_r_. Thus, the change of *E*_w1_ can affect the velocity of DmK‐6AA.

From Fig. S8C, it is seen that increasing and decreasing *E*_w2_ by 1*k*_B_ *T* increases and decreases the run length, respectively, by about 20–60% under no and backward loads, whereas changing *E*_w2_ by 1*k*_B_ *T* has a smaller effect (<20%) on the run length under forward load. This can be understood as follows. Under no and backward loads, the run length is determined largely by the dissociation occurring during Period II, with the dissociation rate depending approximately exponentially on *E*_w2_. Under the forward load, the run length is determined mainly by the dissociation occurring during Period I, which is independent of *E*_w2_, and the dissociation occurring during Period II has a small contribution to the overall dissociation. As expected, under forward and low backward loads, changing *E*_w2_ by 1*k*_B_ *T* has nearly no effect on the velocity, because the dissociation has nearly no effect on the velocity. However, under a large backward load (e.g. *F*_*x*_ = –4.3 pN), increasing and decreasing *E*_w2_ by 1*k*_B_ *T* increases and decreases the velocity by about 4% and 25%, respectively. This is because decreasing *E*_w2_ increases the probability of the leading ADP–head detaching from MT before ADP release, thus increasing the probability of backward stepping under the large backward load.

From Fig. S8D, it is seen that changing *k*_NL_ by 10% has only a slight effect on the velocity, but increasing and decreasing *k*_NL_ results in an evident but small (< 6%) increase and decrease in the run length, respectively. This can be understood as follows. Since *k*_NL_ is large and non‐rate limiting, changing its value by 10% has only a slight effect on the ATPase rate and thus has only a slight effect on the velocity. The occurrence probability of Period I increases with the increase in ratio of *k*_c_ to *k*_NL_. Thus, increasing *k*_NL_ decreases the occurrence probability of Period I, decreasing the dissociation probability and increasing the run length.

From Fig. S8E, it is seen that increasing and decreasing *k*_c_ by 10% results in a small (< 3%) increase and decrease in the velocity, respectively. This is easily understandable, because increasing *k*_c_ increases the ATP rate and thus increases the velocity. On the contrary, increasing and decreasing *k*_c_ by 10% decreases and increases the run length, respectively, by about 10%. This can be understood as follows. The occurrence probability of Period I increases with the increase in ratio of *k*_c_ to *k*_NL_, and the occurrence probability of Period II increases with the increase in ratio of *k*_c_ to *k*_–D_. Thus, increasing *k*_c_ increases the occurrence probabilities of both Period I and Period II, increasing the dissociation probability and decreasing the run length.

## Concluding remarks

We present an improved model for the processive movement of the dimeric kinesin‐1 molecular motor, with which we study the movement dynamics of the motor, particularly for the run length and velocity, to understand the detailed molecular mechanism of mechanochemical coupling and coordination of the two heads of the dimer during its highly processive movement on MT. The dissociation of the motor from MT occurs during two periods—Period I and Period II—when the motor is in the intermediate or 1HB state. Period I is after P_i_ release occurs in the MT‐bound head prior to its NL docking and before the affinity of the MT‐bound ADP–head for the local MT changes from much weaker *E*_w1_ to weak *E*_w2_ (see inset of Fig. 1), while Period II is when the MT‐bound head is in the ADP state with its weak affinity for the local MT being *E*_w2_. During other periods when there is at least one head in the nucleotide‐free, ATP, or ADP.P_i_ state bound strongly to MT, the dissociation of the motor is negligible. In a mechanochemical coupling, both Period I and Period II occur rarely, resulting in a very low probability of dissociation and thus a high processivity.

We provide a quantitative explanation of the puzzling single‐molecule data on the run length *versus* external load acting on the coiled‐coil stalk, revealing the origin of the dramatically asymmetric character of the run length with respect to the direction of the load and the run length dropping sharply under even a small forward load. The origin is due to the asymmetric occurrence probability of Period I, which is in turn due to the fact that the P_i_ release rate in the MT‐bound head is much larger when its NL is driven in the forward direction under the forward load than when the NL is not in the forward direction under the backward load. The contrary features of the run length for different types of kinesin‐1 (*Drosophila* and human kinesins) with extensions of their NLs compared with those without extension of the NLs are explained quantitatively. The origin of the contrary features is as follows. For *Drosophila* kinesin with *E*_w2_ = 40*k*_B_ *T*, the dissociation during Period II makes the main contribution to the overall dissociation under no or backward load. By contrast, for human kinesin with *E*_w2_ = 43*k*_B_ *T*, the dissociation during Period II makes a smaller contribution than Period I to the overall dissociation under no load. The extension of the NLs decreases the ADP‐release rate from the leading head. This increases the occurrence probability of Period II but decreases the occurrence probability of Period I, thus resulting in the decrease in the processivity for *Drosophila* kinesin but a slight increase of the processivity for human kinesin. In addition, the single‐molecule data on durations of the 1HB and 2HB states during processive movement are explained quantitatively. The single‐molecule data showing the enhancement of the run length by the addition of phosphate in solution are explained well. The computational data on other aspects of the movement dynamics such as the force dependence of velocity are also in quantitative agreement with the available experimental data. In a word, the above diverse, puzzling, and contrary experimental data are explained quantitatively and consistently with the same set of parameter values (Table 1).

Moreover, we predict that in sharp contrast to the case under the forward load acting on the coiled‐coil stalk, the run length only decreases mildly with the forward load acting on one head of the dimer, with the latter case giving an opposite asymmetry of the run length with respect to the direction of the load compared to the former case (Fig. 7A). These predictions can be tested easily by future single‐molecule optical trapping assays.

## Author contributions

SG wrote programs, performed simulations, analyzed the data, and reviewed the manuscript. XS and PW analyzed the data and reviewed the manuscript. PX proposed the model, organized the research, analyzed the data, and wrote the manuscript.

## Supporting information


**Appendix S1.** Extended Materials and Methods.
**Fig. S1.** Force–extension relation of the neck linker of DmK‐WT head.
**Fig. S2.** Force–extension relation of the neck linker of DmK‐6AA head.
**Fig. S3.** Force–extension relation of the neck linker of HsK‐CL head.
**Fig. S4.** Force–extension relation of the neck linker of HsK‐CL‐6AA head.
**Fig. S5.** Effects of the presence of additional P_i_ in solution on run length and velocity of DmK‐WT at saturating ATP.
**Fig. S6.** Effect of the neck linker docking on the velocity of kinesin‐1.
**Fig. S7.** Effect of the solution viscosity η on the velocity of kinesin‐1.
**Fig. S8.** The sensitivity of the calculated run length and velocity to the relevant parameters for DmK‐WT.
