# Supplementary material for: Processivity of dimeric kinesin‐1 molecular motors
Source: FEBS Open Bio. 2018 Jul 20;8(8):1332–51. doi: 10.1002/2211-5463.12486 (PMC6070657; doi:10.1002/2211-5463.12486)
Supplement: Supplementary file 1 — Appendix S1. Extended Materials and Methods. Fig. S1. Force–extension relation of the neck linker of DmK‐WT head. Fig. S2. Force–extension relation of the neck linker of DmK‐6AA head. Fig. S3. Force–extension relation of the neck linker of HsK‐CL head. Fig. S4. Force–extension relation of the neck linker of HsK‐CL‐6AA head. Fig. S5. Effects of the presence of additional Pi in solution on run length and velocity of DmK‐WT at saturating ATP. Fig. S6. Effect of the neck linker docking on the velocity of kinesin‐1. Fig. S7. Effect of the solution viscosity η on the velocity of kinesin‐1. Fig. S8. The sensitivity of the calculated run length and velocity to the relevant parameters for DmK‐WT. [file FEB4-8-1332-s001.pdf]

Supplementary Information

Processivity of dimeric kinesin-1 molecular motors

Si-Kao Guo [1,2], Xiao-Xuan Shi [1,2], Peng-Ye Wang [1,2], Ping Xie [1,2,*]

[1] *Key Laboratory of Soft Matter Physics, Beijing National Laboratory for Condensed Matter Physics, Institute of Physics, Chinese Academy of Science, Beijing 100190, China,*
[2] *School of Physical Sciences, University of Chinese Academy of Sciences, Beijing 100049, China*

*Corresponding author:
E-mail address: pxie@aphy.iphy.ac.cn

Appendix S1. Extended Material and Methods

S1. Potential of interaction between one kinesin head and MT in a mechanochemical coupling cycle

Based on argument (i) (see main text), we take the interaction potential of one kinesin head with MT as described as follows. In nucleotide-free state the kinesin head binds strongly to MT, with the interaction potential being written as $V_S(x,y,z,\alpha,\theta,\phi)=V_{Sx}(x)V_y(y)V_z(z)V_\alpha(\alpha)V_\theta(\theta)V_\phi(\phi)$, where coordinate *oxyz* is defined in Fig. 1, α, θ and ϕ are angles characterizing respectively the nutation, rotation and precession motions (when the kinesin head is in the MT-binding site, α, θ and ϕ correspond to the angles of rotations in *xoz*, *xoy* and *yoz* planes, respectively). Term $V_{Sx}(x) < 0$ (with the maxima equal to zero) represents the interaction potential between the kinesin head and MT along a MT protofilament and is approximately shown in the inset of Fig. 1 (upper panel). The period of $V_{Sx}(x)$, d = 8.2 nm, is equal to the distance between two successive binding sites on MT filaments. To be consistent with that for the case of the monomeric KIF1A [1], we take $V_{Sx}(x)$ in one periodicity having an asymmetric form here, with asymmetric ratio d_1/d_2 = 3/5. It should be mentioned that taking other forms for $V_{Sx}(x)$ (including the symmetric one) has little effect on the results for the dimer [2]. Terms $V_y(y)\equiv\exp\left[-(y-y_0)/A_y\right]$ and $V_z(z)\equiv\exp\left(-\left|z-z_0\right|/A_z\right)$ denote the potential changes in the vertical and horizontal

directions, respectively, with A_y and A_z characterizing the interaction distances. Note that, due to the steric restriction of MT, the position of the kinesin head is confined to the region $y \geq y_0$. Terms $V_\alpha(\alpha) \equiv \exp\left(-|\alpha-\alpha_0|/A_\alpha\right)$, $V_\theta(\theta) \equiv \exp\left(-|\theta-\theta_0|/A_\theta\right)$ and $V_\phi(\phi) \equiv \exp\left(-|\phi-\phi_0|/A_\phi\right)$ denote the potential changes arising from the head rotations, with A_α, A_θ and A_ϕ characterizing the interaction distances. Here, we define $y_0 = z_0 = \alpha_0 = \theta_0 = \phi_0 = 0$ in the MT-binding state. These potential changes $V_y(y)$, $V_z(z)$, $V_\alpha(\alpha)$, $V_\theta(\theta)$ and $V_\phi(\phi)$ are similar to the Morse potential form that describes the van der Waals interaction. To be consistent with the Debye length that is in the order of 1 nm in solution, we take $A_y = A_z = 1$ nm and $r_{head}A_\alpha = r_{head}A_\theta = r_{head}A_\phi$ = 1 nm, where the kinesin head is approximately a sphere of radius r_{head} = 2.5 nm. After ATP binding and then hydrolysis to ADP.Pi the kinesin head remains bound strongly to MT, with the interaction potential still being approximately described by $V_S(x, y, z, \alpha, \theta, \phi)$.

Immediately after Pi release, the interaction potential becomes one that can be written as $V_W(x, y, z, \alpha, \theta) = V_{Wx1}(x)V_y(y)V_z(z)V_\alpha(\alpha)V_\theta(\theta)V_\phi(\phi)$, with $V_{Wx1}(x) < 0$ being approximately shown in the inset of Fig. 1 (middle panel) and $V_y(y)$, $V_z(z)$, $V_\alpha(\alpha)$, $V_\theta(\theta)$ and $V_\phi(\phi)$ being the same as those defined above. Note that immediately after Pi release the binding affinity of kinesin for the local MT-binding site at which the ADP.Pi-kinesin has just bound , E_{w1}, is weaker than at other MT-binding sites, E_{w2}. After a period of time, t_r, the affinity of the local MT-binding site for ADP-head relaxes to the normal value and the interaction potential becomes $V_W(x, y, z, \alpha, \theta) = V_{Wx2}(x)V_y(y)V_z(z)V_\alpha(\alpha)V_\theta(\theta)V_\phi(\phi)$, with $V_{Wx2}(x)$ being shown approximately in the inset of Fig. 1 (lower panel). Note that the weak binding affinity of ADP-kinesin for MT, E_{w2}, is smaller than the strong binding affinity of nucleotide-free, ATP- or ADP.Pi-kinesin for MT, E_S.

S2. Potential of the effect of neck linker docking to the motor domain of the MT-bound head on the movement of the other ADP-head

Structural studies showed that the N-terminal strand of motor domain, $\beta 0$, contributes to the docking of the strand $\beta 9$ of the neck linker (NL) by forming a cover-neck bundle (CNB) [3]. Thus, it is assumed that only when $\beta 0$ and $\beta 9$ are

in proximity can CNB form. As a result, the effect of the NL docking to the MT-bound head on the motion of the detached head relative to the MT-bound head can be approximately characterized by a potential $V_{NL}(x)$, which has the form

$$V_{NL}(x) = E_{NL}, \qquad x \le 0, \tag{S1}$$

$$V_{NL}(x) = -E_{NL}(x-1), \quad 0 < x \le 1, \tag{S2}$$

$$V_{NL}(x) = 0, \qquad x > 1, \tag{S3}$$

where it is considered that the MT-bound head is at position (x, y, z) = (0, 0, 0), and E_{NL} is the free energy change of the NL docking. This form of $V_{NL}(x)$ implies that when the detached head is in the range of $x \le 0$ relative to the MT-bound head, $\beta 9$ is far away from $\beta 0$ and CNB is unable to from, when $x > 0$, $\beta 0$ and $\beta 9$ are in proximity and CNB can form, and when $x > 1$, CNB is formed and thus the NL docking is completed, allowing the detached head to move forward freely but preventing it from moving backward.

S3. Potential of interaction between two kinesin heads

We take the potential of interaction between two kinesin heads with the NL of the MT-bound head being undocked having the following form

$$\begin{aligned} V_I(x,y,z,\alpha,\theta,\phi) = -E_{I1}\exp\left(-\frac{\sqrt{(x-x_1)^2+(y-y_1)^2+(z-z_1)^2}}{A_r}\right) \\ \times\exp\left(-\frac{|\alpha-\alpha_1|}{A_\alpha}\right)\exp\left(-\frac{|\theta-\theta_1|}{A_\theta}\right)\exp\left(-\frac{|\phi-\phi_1|}{A_\phi}\right) \end{aligned}, \tag{S4}$$

where (x, y, z) is the center-of-mass coordinate of the detached ADP-head relative to that of the MT-bound head (which is taken as the origin of the coordinate) during one stepping period, (x_1, y_1, z_1) is the position of the detached ADP-head in the intermediate state, α_1, θ_1 and ϕ_1 are the nutation, rotation and precession angles of the detached ADP-head in the intermediate state, $E_{I1} > 0$ is the strong interaction strength and A_r = 1 nm characterizes the interaction distance. Based on the available structural data [4], we take $\alpha_1 = 180°$, $\theta_1 = -80°$ and ϕ_1 = 0 in the calculation, thus

giving (x_1, y_1, z_1) = $2r_{head} \times (\cos 80^\circ, \sin 80^\circ, 0)$. Note that taking other values of α_1, θ_1 and ϕ_1 has nearly no effect on our results given in this work. When the NL of the MT-bound head is docked, the potential is still described by Eq. (S4), but with E_{I1} being replaced with E_{I2} ($E_{I2} < E_{I1}$).

S4. Interaction between the neck linker and kinesin head

To determine the interaction between the NL and kinesin head, we should determine the elasticity of the NL by using all-atom MD simulations (see Section S5), as used elsewhere [5,6]. Here, we take the NL of wild-type *Drosophila* kinesin as an example to describe the simulation procedure. We take residues 324 through 338 from the structural data (PDB 3KIN), where residues 325 – 338 constitute the NL. We adjust the line connecting the alpha carbon (CA) atom of residue 324 and that of residue 338 along a given direction. We fix the CA atom of the residue 324 and impose a series of constant forces on the CA atom of the residue 338 along the given direction, as done before [7]. We then calculate the distance, r_{NL}, between the two terminal CAs of the NL after reaching the equilibrium for 20 ns. In the literature, the calculated data of the force–extension relation of a flexible peptide were usually fitted by using the worm-like-chain (WLC) model. However, it is noted that the simulated data of the force–extension relation of the kinesin's NL are better fitted by using the exponential function than WLC model, especially at small values of pulling force (see Fig. S1). Thus, here we use the exponential function to fit the simulated data

$$F_{NL}\left(r_{NL}\right) = a\exp\left(br_{NL}\right), \qquad \text{(S5)}$$

where a and b are constants. Then the force on the detached head, which results from the stretched NL, can be calculated by using Eq. (S5).

S5. All-atom MD simulations

The all-atom MD simulations are carried out by using GROMACS5.1 [8] with OPLS-AA/L all-atom force field [9]. To avoid the edge effect, the distance between the peptide of the NL and the boundary of the box is at least 1.5 nm and much longer along the direction of the pulling force to stretch the NL. We add solvent and necessary ions with favorable concentration. Counter-ions are also added to neutralize

the system. All MD simulations are run at 300K and 1 bar. The time step is set as 2 fs, and the output data is updated every ps. All chemical bonds are constrained using LINCS algorithm [10]. The short-range electrostatics interaction and the cutoff for van der Waals interaction is set as 1 nm. Velocity-rescaling temperature coupling [11] and Berendsen pressure coupling [12] are used. The energy minimization is performed using the steepest descent method. Before the dynamic simulations, the systems are equilibrated successfully for 2 ns at 300 K and 1 bar pressure in the NVT ensemble and NPT ensemble, respectively. For calculations of the force–extension relation of the NL, after a 20-ns constant force–extension simulation, the distance between the two terminal CAs of the NL is extracted from the output trace files using the VMD1.9.2 [13].

S6. Equation for kinesin dimer under an external force acting on the bead attached to one head

Consider a micro-meter-sized bead with diameter of $2R_{bead}$ attached to one head of the dimer and an external force acting on the bead. For this case, the equations for the translation and rotation of the ADP-head, which is attached by the bead, relative to the other head binding fixedly to MT at position (0,0,0) can be described by following Langevin equations

$$\Gamma_x \frac{\partial x}{\partial t} = -\frac{\partial V_W(x,y,z,\alpha,\theta)}{\partial x} - \frac{\partial V_I(x,y,z,\alpha,\theta)}{\partial x} - F_{NL}\left(\frac{r}{2}\right)\frac{x}{r} - \frac{\partial V_{NL}(x)}{\partial x} - C\left(x - x_{bead}\right) + \xi_x(t), \quad \text{(S6)}$$

$$\Gamma_y \frac{\partial y}{\partial t} = -\frac{\partial V_W(x,y,z,\alpha,\theta)}{\partial y} - \frac{\partial V_I(x,y,z,\alpha,\theta)}{\partial y} - F_{NL}\left(\frac{r}{2}\right)\frac{y}{r} - C\left(y - y_{bead}\right) + \xi_y(t), \quad \text{(S7)}$$

$$\Gamma_x^{(bead)} \frac{\partial x_{bead}}{\partial t} = C\left(x - x_{bead}\right) + F_x + \xi_X(t), \quad \text{(S8)}$$

$$\Gamma_y^{(bead)} \frac{\partial y_{bead}}{\partial t} = C\left(y - y_{bead}\right) + F_y + \xi_Y(t), \quad \text{(S9)}$$

$$\Gamma_z \frac{\partial z}{\partial t} = -\frac{\partial V_W(x,y,z,\alpha,\theta,\phi)}{\partial z} - \frac{\partial V_I(x,y,z,\alpha,\theta,\phi)}{\partial z} - F_{NL}\left(\frac{r}{2}\right)\frac{z}{r} + \xi_z(t), \quad \text{(S10)}$$

$$\Gamma_\alpha \frac{\partial \alpha}{\partial t} = -\frac{\partial V_W(x,y,z,\alpha,\theta,\phi)}{\partial \alpha} - \frac{\partial V_I(x,y,z,\alpha,\theta,\phi)}{\partial \alpha} + \xi_\alpha(t), \quad \text{(S11)}$$

$$\Gamma_\theta \frac{\partial \theta}{\partial t} = -\frac{\partial V_W(x,y,z,\alpha,\theta,\phi)}{\partial \theta} - \frac{\partial V_I(x,y,z,\alpha,\theta,\phi)}{\partial \theta} + \xi_\theta(t), \quad \text{(S12)}$$

$$\Gamma_\phi \frac{\partial \phi}{\partial t} = -\frac{\partial V_W(x,y,z,\alpha,\theta,\phi)}{\partial \phi} - \frac{\partial V_I(x,y,z,\alpha,\theta,\phi)}{\partial \phi} + \xi_\phi(t). \tag{S13}$$

where for simplicity but without loss of generality, the motion of the bead in the z-coordinate is not considered, as done in Eqs. (1) – (12). The initial conditions for Eqs. (S6) – (S13) are: $(x_0, y_0, z_0, \alpha_0, \theta_0, \phi_0)$ = ($-d$, 0, 0, 0, 0, 0), $x_{bead0} = -d + F_x/C$ and $y_{bead0} = F_y/C$. For the ADP-head that is not attached by the bead, its translation motion relative to the other head binding fixedly to MT at position (0,0,0) can still be described by Eqs. (S6), (S7) and (S10) but with C = 0 and its rotation can still be described by Eqs. (S11) – (S13). Here, we still consider the linker connecting the head and bead has a length $l = l_{kinesin}$ = 54 nm. Thus, For the longitudinal or x-component of the external force, F_x, the vertical or y-component is still calculated by $F_y = |F_x| \tan\Theta_0$ and $\sin\Theta_0 = R_{bead} / (R_{bead} + l_{kinesin})$, with $2R_{bead}$ = 0.44 μm.

In the intermediate state, where the two ADP-heads bind together strongly, the equations for the translation and rotation of the MT-bound ADP-head relative to MT can be described by following Langevin equations

$$\Gamma_x \frac{\partial x}{\partial t} = -\frac{\partial V_W(x,y,z,\alpha,\theta,\phi)}{\partial x} - C(x - x_{bead}) + \xi_x(t), \tag{S14}$$

$$\Gamma_y \frac{\partial y}{\partial t} = -\frac{\partial V_W(x,y,z,\alpha,\theta,\phi)}{\partial y} - C(y - y_{bead}) + \xi_y(t), \tag{S15}$$

$$\Gamma_x^{(bead)} \frac{\partial x_{bead}}{\partial t} = C(x - x_{bead}) + F_x + \xi_X(t), \tag{S16}$$

$$\Gamma_y^{(bead)} \frac{\partial y_{bead}}{\partial t} = C(y - y_{bead}) + F_y + \xi_Y(t), \tag{S17}$$

$$\Gamma_z \frac{\partial z}{\partial t} = -\frac{\partial V_W(x,y,z,\alpha,\theta,\phi)}{\partial z} + \xi_z(t), \tag{S18}$$

$$\Gamma_\alpha \frac{\partial \alpha}{\partial t} = -\frac{\partial V_W(x,y,z,\alpha,\theta,\phi)}{\partial \alpha} + \xi_\alpha(t), \tag{S19}$$

$$\Gamma_\theta \frac{\partial \theta}{\partial t} = -\frac{\partial V_W(x,y,z,\alpha,\theta,\phi)}{\partial \theta} + \xi_\theta(t), \tag{S20}$$

$$\Gamma_\phi \frac{\partial \phi}{\partial t} = -\frac{\partial V_W(x,y,z,\alpha,\theta,\phi)}{\partial \phi} + \xi_\phi(t), \tag{S21}$$

where, for simplicity of treatment, the head and bead are implicitly considered to be

rigidly connected. The initial conditions for Eqs. (S14) – (S21) are: $(x_0, y_0, z_0, \alpha_0, \theta_0, \phi_0)$ = (0,0,0,0,0,0), $x_{bead0} = F_x/C$ and $y_{bead0} = F_y/C$.

References

[1] P. Xie, S.-X. Dou and P.-Y. Wang, Processivity of single-headed kinesin motors, *Biochimica et Biophysica Acta (BBA) - Bioenergetics*, 2007, **1767**, 1418-1427.

[2] S.-K. Guo, P.-Y. Wang and P. Xie, A model of processive movement of dimeric kinesin, *Journal of Theoretical Biology*, 2017, **414**, 62-75.

[3] W. Hwang, M. J. Lang and M. Karplus, Force generation in kinesin hinges on cover-neck bundle formation, *Structure*, 2008, **16**, 62-71.

[4] M. C. Alonso, D. R. Drummond, S. Kain, J. Hoeng, L. Amos and R. A. Cross, An ATP gate controls tubulin binding by the tethered head of kinesin-1, *Science*, 2007, **316**, 120-123.

[5] Z.-W. Duan, P. Xie, W. Li and P.-Y. Wang, Are coiled-coils of dimeric kinesins unwound during their walking on microtubule? *PLOS ONE*, 2012, **7**, e36071.

[6] Y.B. Fu, Z.F. Wang, P.Y. Wang and P. Xie, Optimal numbers of residues in linkers of DNA polymerase I, T7 primase and DNA polymerase IV. *Scientific Reports*, 2016, **6**, 29125.

[7] V. Hariharan and W. O. Hancock, Insights into the mechanical properties of the kinesin neck linker domain from sequence analysis and molecular dynamics simulations, *Cellular and Molecular Bioengineering*, 2009, **2**, 177-189.

[8] M. J. Abraham, T. Murtola, R. Schulz, S. Páll, J. C. Smith, B. Hess and E. Lindahl, GROMACS: high performance molecular simulations through multi-level parallelism from laptops to supercomputers, *SoftwareX*, 2015, **1–2**, 19-25.

[9] W. L. Jorgensen, D. S. Maxwell and J. Tirado-Rives, Development and testing of the OPLS all-atom force field on conformational energetics and properties of organic liquids, *Journal of the American Chemical Society*, 1996, **118**, 11225-11236.

[10] B. Hess, P-LINCS: A parallel linear constraint solver for molecular simulation, *Journal of Chemical Theory and Computation*, 2008, **4**, 116-122.

[11] G. Bussi, D. Donadio and M. Parrinello, Canonical sampling through velocity rescaling, *The Journal of Chemical Physics*, 2007, **126**, 014101.

[12] H. J. C. Berendsen, J. P. M. Postma, W. F. van Gunsteren, A. DiNola and J. R. Haak, Molecular dynamics with coupling to an external bath, *The Journal of Chemical Physics*, 1984, **81**, 3684-3690.

[13] W. Humphrey, A. Dalke and K. Schulten, VMD - visual molecular dynamics, *Journal of Molecular Graphics*, 1996, **14**, 33-38.

[14] Khalil, A. S., Appleyard, D. C., Labno, A. K., Georges, A., Karplus, M., Belcher, A. M., Hwang, W., Lang, M. J. (2008) Kinesin's cover-neck bundle folds forward to generate force. *Proceedings of the National Academy of Sciences* 105,19247–19252.

[15] Sozański K., Ruhnow, F., Wisniewska, A., Tabaka, M., Diez, S., Holyst, R. (2015) Small Crowders Slow Down Kinesin-1 Stepping by Hindering Motor Domain Diffusion. *Physical Review Letters* 115, 218102.

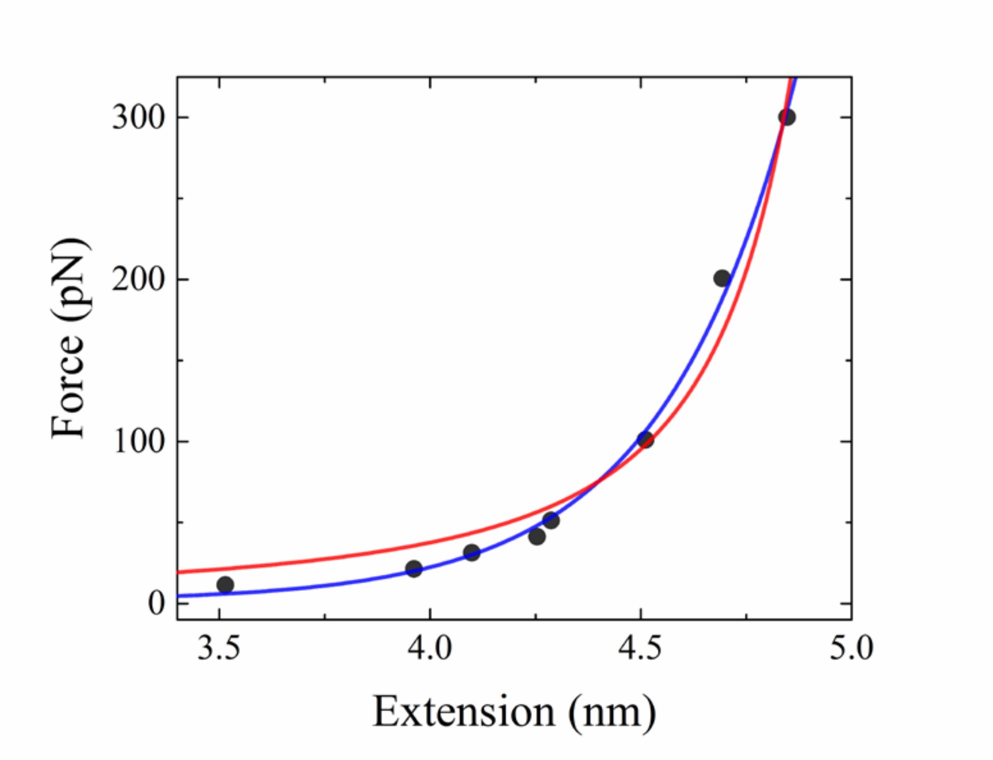

Fig. S1. Force–extension relation of the neck linker of DmK-WT head. Dots represent the results obtained by using all-atom MD simulations. Red lines is the fit curve with WLC model, $f(x) = k_B T / (4L_p)\left[(1 - x/L_c)^{-2} - 1 + 4x/L_c\right]$, where the contour length L_c = 5.26 nm and the persistence length L_p = 0.56 nm. Blue line is the fit curve with Eq. (S5), $F_{NL}(r_{NL}) = a\exp(br_{NL})$, with a = 7.064×10^{-5} pN and b = 3.148 nm^{-1}.

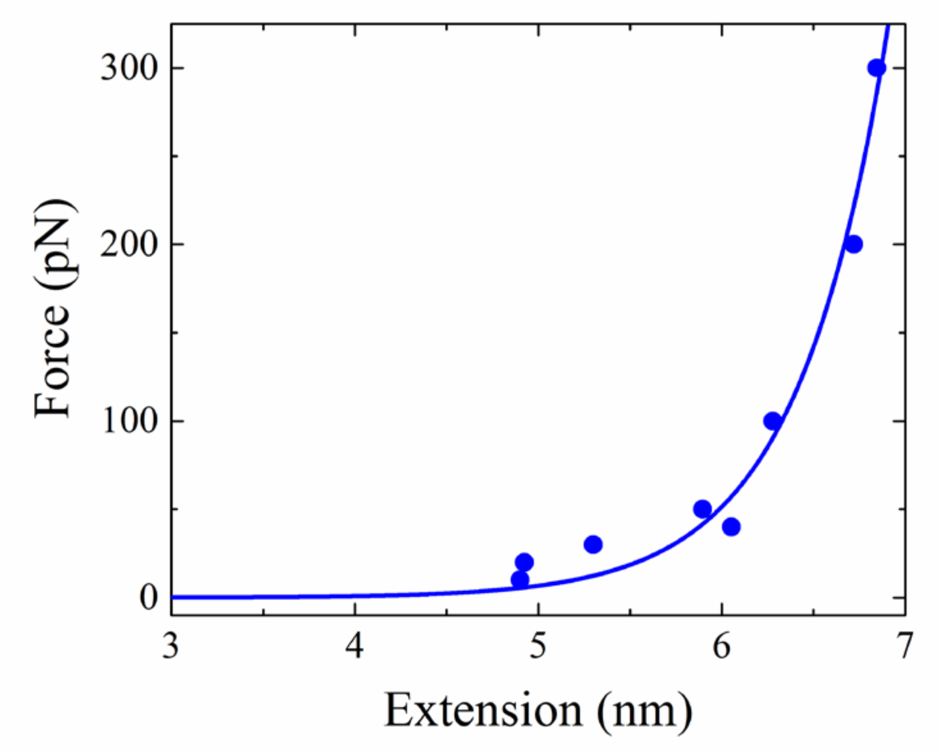

Fig. S2. Force–extension relation of the neck linker of DmK-6AA head. Dots represent the results obtained by using all-atom MD simulations. Line is the fit curve with Eq. (S5), $F_{NL}(r_{NL}) = a\exp(br_{NL})$, with a = 2.623×10^{-4} pN and b = 2.031 nm^{-1}.

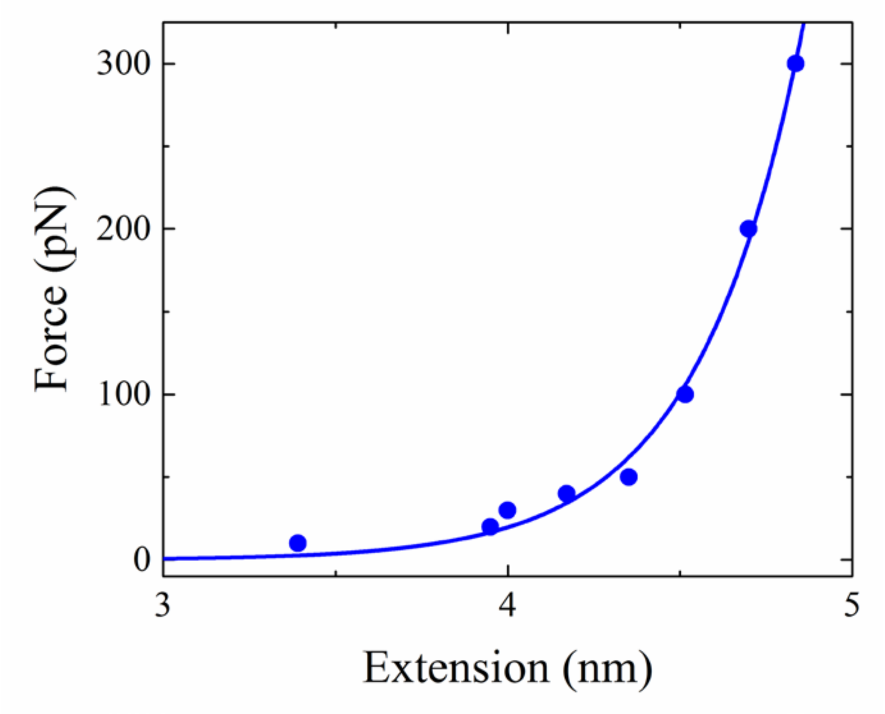

Fig. S3. Force–extension relation of the neck linker of HsK-CL head. Dots represent the results obtained by using all-atom MD simulations. Line is the fit curve with Eq. (S5), $F_{NL}(r_{NL}) = a\exp(br_{NL})$, with a = 4.226×10^{-5} pN and b = 3.263 nm^{-1}.

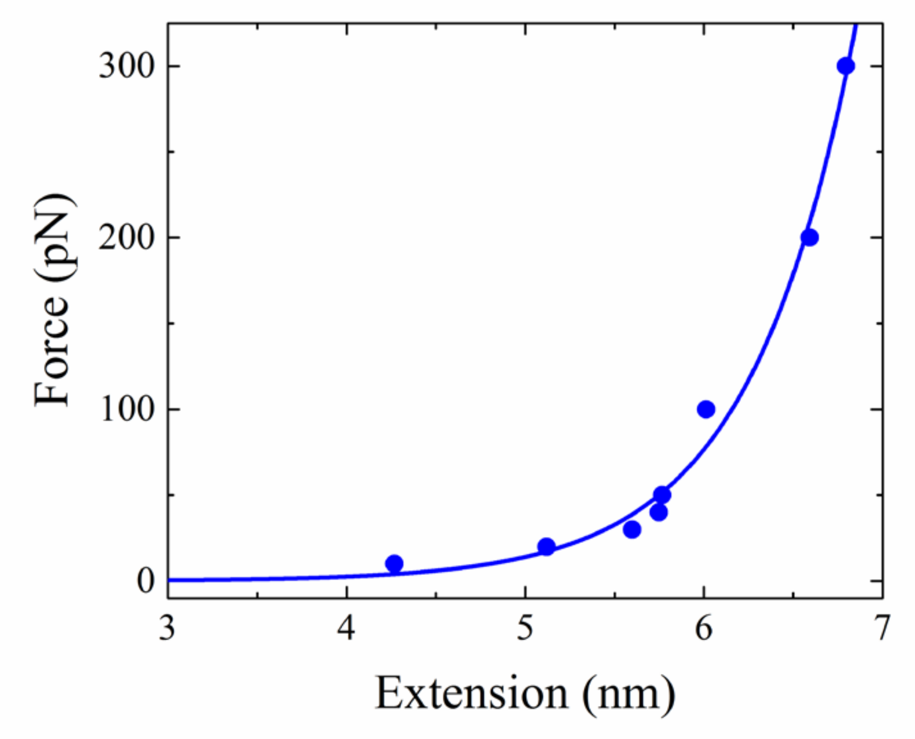

Fig. S4. Force–extension relation of the neck linker of HsK-CL-6AA head. Dots represent the results obtained by using all-atom MD simulations. Line is the fit curve with Eq. (S5), $F_{NL}\left(r_{NL}\right)=a\exp\left(br_{NL}\right)$, with a = 2.936×10^{-3} pN and b = 1.695 nm^{-1}.

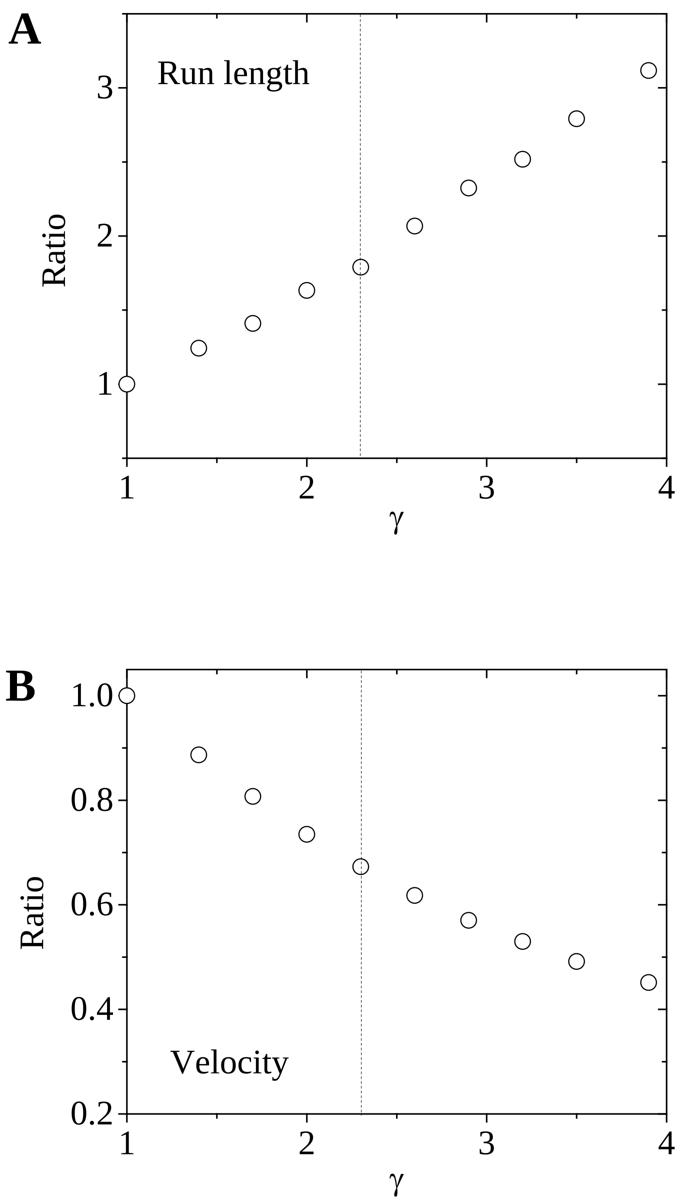

Fig. S5. Effects of the presence of additional Pi in solution on run length and velocity of DmK-WT at saturating ATP. (A) Ratio of the calculated run length in the presence of additional Pi to that without the additional Pi at F_x = 4 pN as a function of γ. The vertical broken line corresponds to γ = 2.3. (**B)** Ratio of the calculated velocity in the presence of additional Pi to that without the additional Pi at F_x = 4 pN as a function of γ. The vertical broken line corresponds to γ = 2.3.

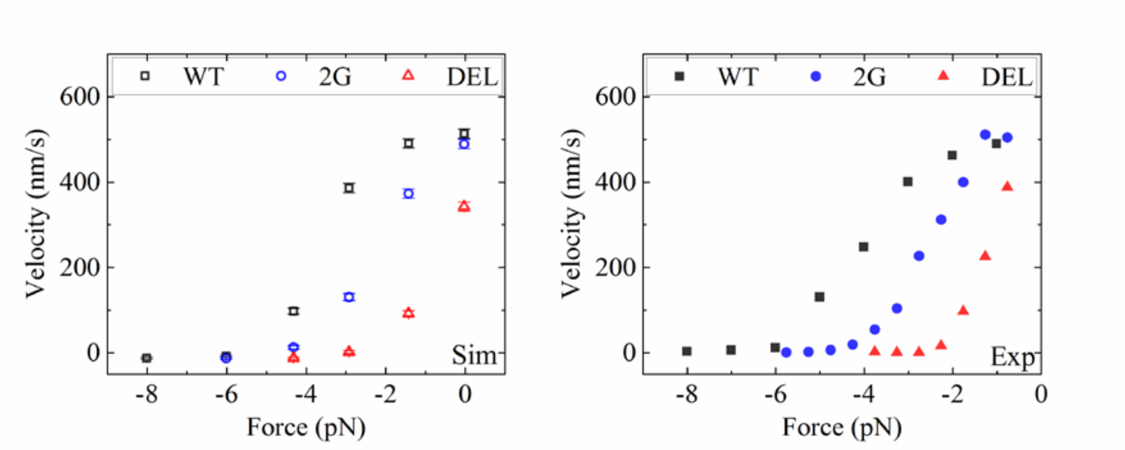

Fig. S6. Effect of the neck linker docking on the velocity of kinesin-1. The panels show the velocity versus longitudinal component (F_x) of the external force for WT, 2G-mutant and DEL. WT refers to the wild-type kinesin-1, 2G-mutant refers to mutant kinesin-1 with A9G and S12G mutations in the N-terminal cover strand that is responsible for the NL docking, and DEL refers to mutant kinesin-1 lacking the entire N-terminal cover strand, as used in the single molecule experiments of Khalil et al. [14]. Here it is considered that the NL docking still occurs in the mutant heads, weakening the interaction between the two heads, but the mutations reduce the NL-docking energy E_{NL}. Values of parameters are taken as follows: $E_{NL} = 4.5k_BT$ for WT, $E_{NL} = 2.5k_BT$ for 2G-mutant and $E_{NL} = 0$ for DEL; other parameters are the same as those for DmK-WT in Fig. 2 except $k_c = 82\ \text{s}^{-1}$. Left panel corresponds to our simulation results and the right panel corresponds to the experimental data taken from Khalil et al. [14].

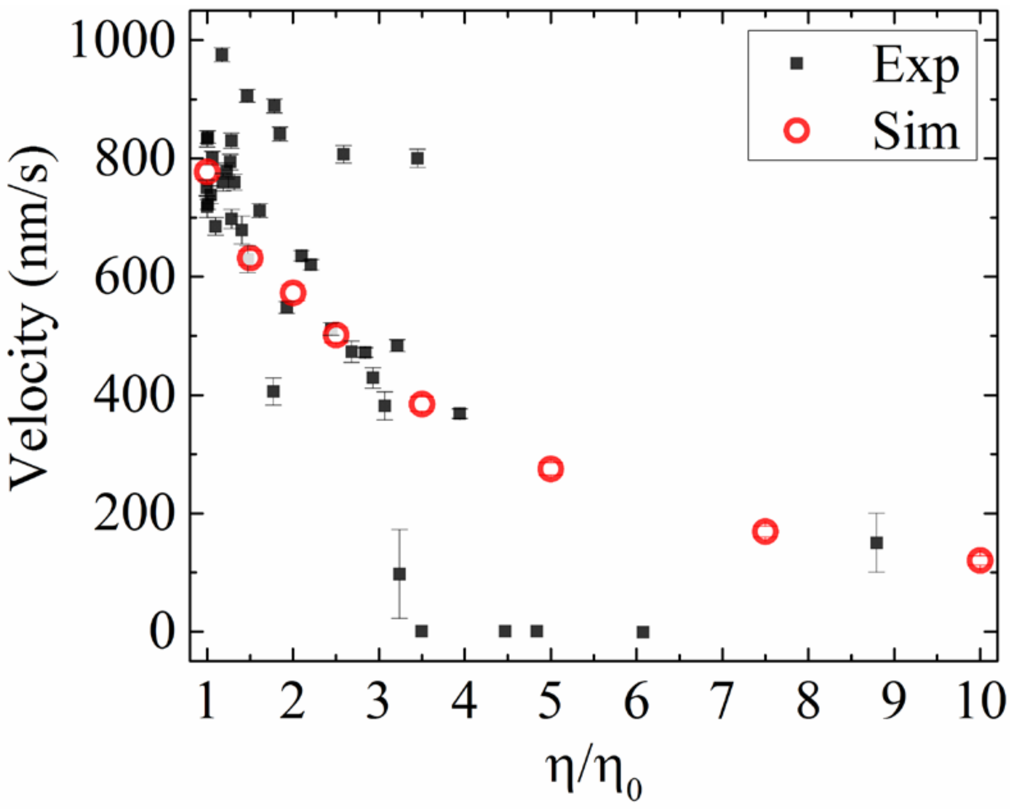

Fig. S7. Effect of the solution viscosity η on the velocity of kinesin-1. The panel shows the velocity versus η/η_0 , where η_0 is the solution viscosity in the vicinity of MT. In the experiments of Sozański et al. [15], no bead is attached to the coiled-coil. This is equivalent to taking spring constant, C = 0, for the connection between the bead and C-terminus ends of the NLs in our calculations using Eqs. (1) – (12). Values of parameters are the same as those for HsK-CL in Fig. 4 except k_c = 240 s^{-1}. Unfilled symbols are calculated data, and filled symbols are experimental data taken from Sozański et al. [15].

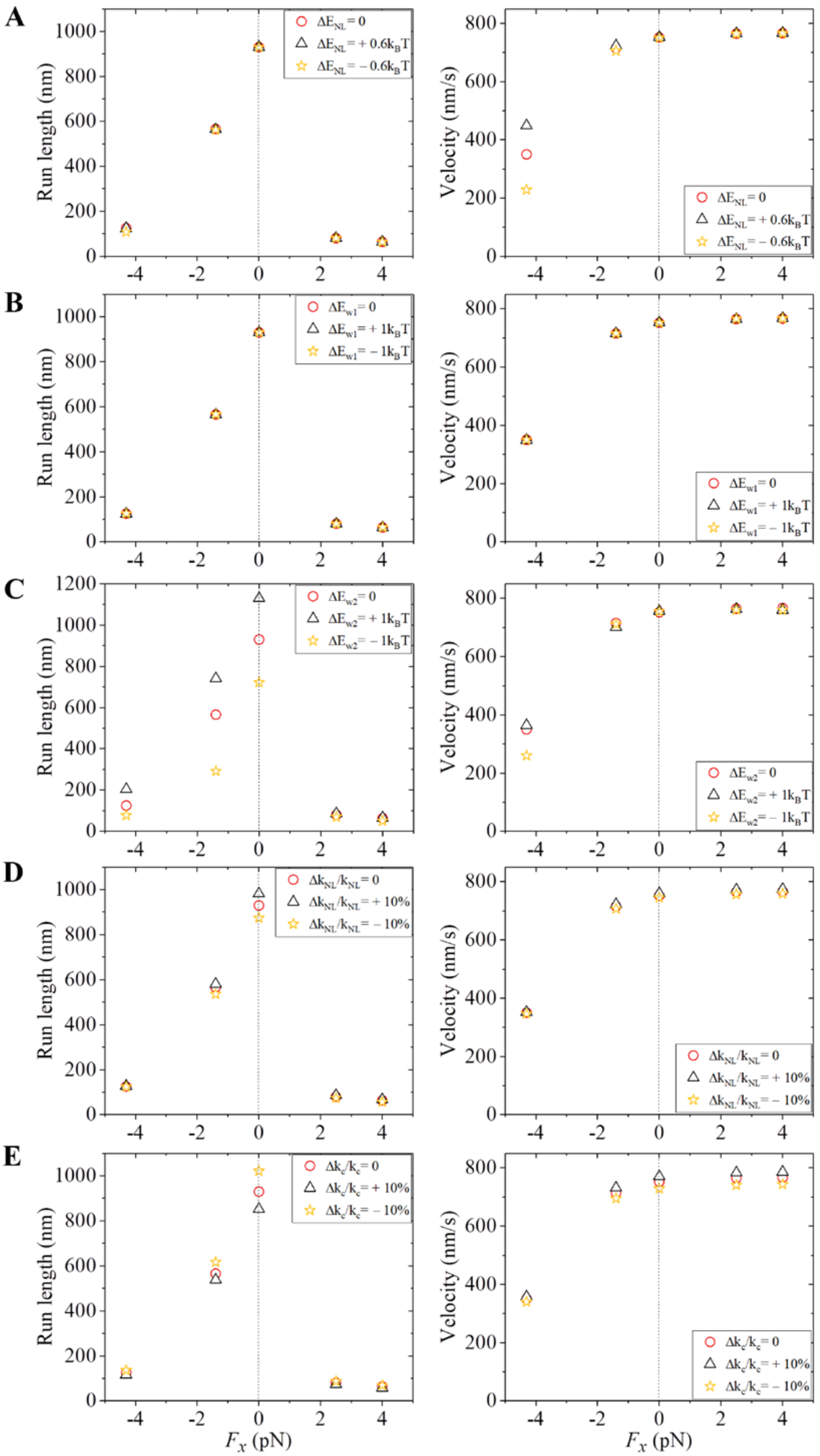
A
B
C
D
E
Run length (nm)
Velocity (nm/s)
ΔE_NL = 0
ΔE_NL = + 0.6k_BT
ΔE_NL = – 0.6k_BT
ΔE_w1 = 0
ΔE_w1 = + 1k_BT
ΔE_w1 = – 1k_BT
ΔE_w2 = 0
ΔE_w2 = + 1k_BT
ΔE_w2 = – 1k_BT
Δk_NL/k_NL = 0
Δk_NL/k_NL = + 10%
Δk_NL/k_NL = – 10%
Δk_c/k_c = 0
Δk_c/k_c = + 10%
Δk_c/k_c = – 10%
F_x (pN)

Fig. S8. The sensitivity of the calculated run length and velocity to the relevant parameters for DmK-WT. Left panels are results of run length versus longitudinal component (F_x) of the external force, while right panels are results of velocity versus F_x. **(A)** Effects of changing E_{NL}. Circles are data calculated with parameter values given in Table 1. Triangles and stars are data calculated with value of E_{NL} increased and decreased by $0.6k_BT$, respectively, while other parameter values are kept unchanged. **(B)** Effects of changing E_{w1}. Circles are data calculated with parameter values given in Table 1. Triangles and stars are data calculated with value of E_{w1} increased and decreased by $1k_BT$, respectively, while other parameter values are kept unchanged. **(C)** Effects of changing E_{w2}. Circles are data calculated with parameter values given in Table 1. Triangles and stars are data calculated with value of E_{w2} increased and decreased by $1k_BT$, respectively, while other parameter values are kept unchanged. **(D)** Effects of changing k_{NL}. Circles are data calculated with parameter values given in Table 1. Triangles and stars are data calculated with value of k_{NL} increased and decreased by 10%, respectively, while other parameter values are kept unchanged. **(E)** Effects of changing k_c. Circles are data calculated with parameter values given in Table 1. Triangles and stars are data calculated with value of k_c increased and decreased by 10%, respectively, while other parameter values are kept unchanged.
